# Microcystin-LR ameliorates pulmonary fibrosis via modulating CD206^+^ M2-like macrophage polarization

**DOI:** 10.1038/s41419-020-2329-z

**Published:** 2020-02-19

**Authors:** Jie Wang, Lizhi Xu, Zou Xiang, Yan Ren, Xiufen Zheng, Qingya Zhao, Qunzhi Zhou, Yuefen Zhou, Lin Xu, Yaping Wang

**Affiliations:** 1 Department of Medical Genetics, Nanjing University School of Medicine, Nanjing, 210093 China; 2 Jiangsu Key Laboratory of Molecular and Translational Cancer Research, Jiangsu Cancer Hospital, Jiangsu Institute of Cancer Research, The Affiliated Cancer Hospital of Nanjing Medical University, Nanjing, 210009 China; 3 Jiangsu Key Laboratory of Molecular Medicine, Nanjing University School of Medicine, Nanjing, 210093 China; 4 Department of Health Technology and Informatics, Faculty of Health and Social Sciences, The Hong Kong Polytechnic University, Hung Hom, Kowloon, Hong Kong China

**Keywords:** Respiratory tract diseases, Translational research

## Abstract

Idiopathic pulmonary fibrosis (IPF) is a group of chronic interstitial pulmonary diseases characterized by myofibroblast proliferation and extracellular matrix deposition with limited treatment options. Based on our previous observation, we hypothesized microcystin-leucine arginine (LR), an environmental cyanobacterial toxin, could potentially suppress pulmonary fibrosis. In this study, we first demonstrated that chronic exposure of microcystin-LR by oral for weeks indeed attenuated the pulmonary fibrosis both on bleomycin-induced rat and fluorescein isothiocyanate-induced mouse models. Our data further indicated that treatment with microcystin-LR substantially reduced TGF-β1/Smad signaling in rat pulmonary tissues. The experiments in vitro found that microcystin-LR was capable of blocking epithelial–mesenchymal transition (EMT) and fibroblast–myofibroblast transition (FMT) through suppressing the differentiation of CD206^+^ macrophages. Mechanically, microcystin-LR was found to bind to glucose-regulated protein 78 kDa (GRP78) and suppress endoplasmic reticulum unfolded protein response (UPR^ER^) signaling pathways. These events led to the modulation of M2 polarization of macrophages, which eventually contributed to the alleviation of pulmonary fibrosis. Our results revealed a novel mechanism that may account for therapeutic effect of microcystin-LR on IPF.

## Introduction

Idiopathic pulmonary fibrosis (IPF), a disease of unknown etiology, is characterized by chronic inflammation, myofibroblast proliferation and exaggerated extracellular matrix (ECM) accumulation, which leads to a progressive decline of lung function with limited therapeutic options^1–4^. The pathogenesis of IPF is currently assumed to be the recurrent or persistent microinjuries to the pulmonary alveolus, which drives microenvironmental changes and provokes a dysregulated tissue repair process^5–7^. The nonspecific insults lead to the destruction of alveolar architecture and induction of macrophage migration and polarization^8–10^. The classical activated macrophages produce anti-microbial mediators, but the macrophage response can be converted from a pro-inflammatory phenotype (M1) to an alternatively activated condition that exhibits an anti-inflammatory phenotype (M2). The alternatively activated macrophages can trigger a pathologic fibrotic-repair mechanism if this irritant persists^11–13^. The pro-fibrotic roles of alternatively activated macrophages are mainly associated with recruitment and proliferation of fibroblasts, and induction of epithelial to mesenchymal transition (EMT) and fibroblast to myofibroblast transition (FMT) through the secretion of fibrogenic mediators, in particular TGF-β1^14,15^. Myofibroblasts are widely believed as the principal effector cells responsible for fibrosis.

Exaggerated TGF-β1 signaling is one of the most studied mechanisms in IPF. When alveolar epithelium is injured, TGF-β1 arisen from infiltrating macrophages and activated fibroblast proliferation enhances synthesis of α smooth muscle actin (αSMA) and collagen, and promotes EMT and FMT by Smad signaling pathway^16,17^. Increased abundance of ECM and enhanced stiffness of lung tissues, which leads to disturbance of the homeostatic microenvironment and dysfunction of pulmonary alveolar surfactant, is a fatal hallmark in the pathogenesis of IPF^18^. Targeting TGF-β1 signaling is thus considered as a therapeutic strategy for IPF, but no convincing treatment efficacy has been obtained^19^.

Microcystins produced by cyanobacteria are a group of cyclic compounds containing seven peptide-linked amino acids. More than 80 different structural analogues of microcystin have been identified. Of these, substitutions of the variable l-amino acids at positions 2 and 4 give rise to at least 21 known microcystin analogues. Microcystin-leucine arginine (LR) displays l-leucine and l-arginine at the two positions, respectively, and is the most abundant toxicant among microcystin variants. Microcystin-LR is broadly reported to pose a threat to aquatic animals and humans^20,21^.

We previously explored the genetic toxicity of microcystin-LR in mice following oral exposure for twelve months^22^. In the following experiments, we unexpectedly found a lower expression of TGF-β1 in the lung tissues after a chronic exposure to microcystin-LR (data not published) and therefore hypothesized a possible suppressive effect of microcystin-LR on fibrosis. In the current study, we have confirmed an anti-fibrotic activity of microcystin-LR through antagonizing macrophage polarization to M2-like phenotype in the animal models and revealed a novel mechanism accounting for therapeutic effect of microcystin-LR on pulmonary fibrosis. To the best of our knowledge, such an anti-fibrotic activity of microcystin-LR has not been previously revealed.

## Results

### Microcystin-LR exerts a therapeutic effect on pulmonary fibrosis in model animals

**Fig. 1:**
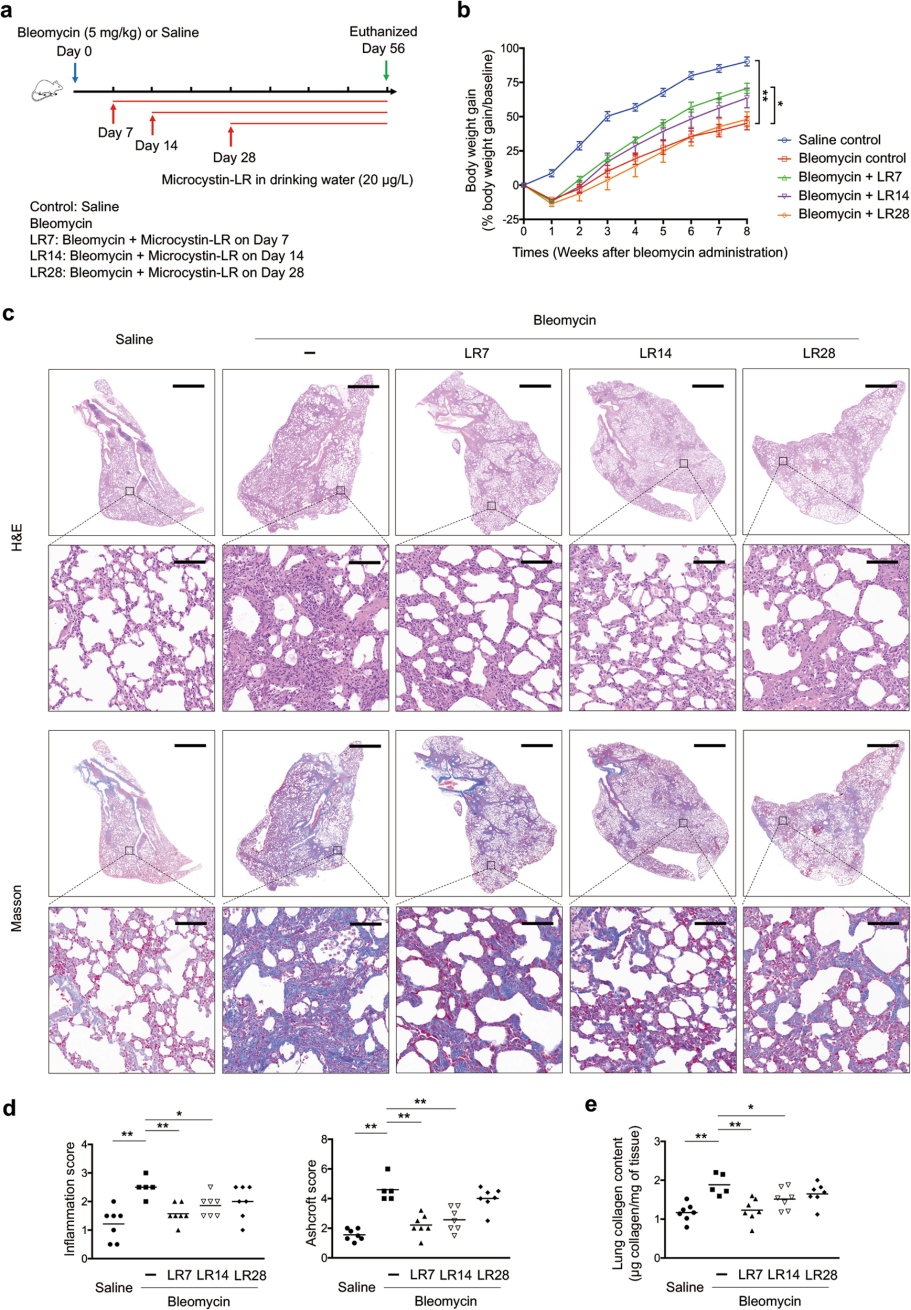
Microcystin-LR exerts an anti-fibrotic activity in rats with bleomycin-induced pulmonary fibrosis. Rats were intratracheally instilled with a single dose of bleomycin (5.0 mg/kg) on day 0 and received microcystin-leucine arginine (LR) (20 μg/L) in drinking water starting on day 7 (LR7), 14 (LR14) or 28 (LR28). Rats were euthanized and samples were collected for analysis day 56 after receiving bleomycin intratracheal instillation. **a** Schematic representation of experimental design is shown. Data of two rats in Bleomycin group were excluded for further analysis due to animal dead. **b** Body weight gain after bleomycin treatment was calculated and shown as percentage increase over the baseline body weight. Data are expressed as mean ± SEM. *P < 0.05, **P < 0.01 determined by two-way ANOVA with Student–Newman–Keuls (S–N–K) post hoc analysis (treatment effect *F*_4,28_ = 15.299, *P* < 0.000; time effect *F*_7,28_ = 478.774, *P* < 0.000; interaction effect *F*_28,28_ = 2.814, *P* < 0.000). **c** Lung tissue sections were prepared and subjected to H&E staining and Masson’s trichrome staining. Scale bar: 2000 μm and 100 μm (insets). **d** The development of lesions revealed by H&E staining (left panel, *F*_4,28_ = 6.434, *P* = 0.001) and Masson’s trichrome (right panel, *F*_4,28_ = 29.255, *P* < 0.000) was scored by pathologists blind to the study design. **e** Lung tissue collagen content was determined by hydroxyproline analysis (*F*_4,28_ = 6.680, *P* = 0.001). *P < 0.05, **P < 0.01 determined by one-way ANOVA with S-N-K post hoc analysis (**d, e**). *n* = 5 (bleomycin) or *n* = 7 (all the other treatments) rats per group.

To investigate the possible effect of microcystin-LR on progressive fibrosis, we exposed rats to microcystin-LR through drinking water starting on days 7 (LR7), 14 (LR14) or 28 (LR28) after intratracheal administration of bleomycin, corresponding to the early phase, transitional phase of inflammation/fibrosis, and the late stage with increased deposition of lung collagen, respectively (Fig. 1a). Fibrosis induction resulted in body weight losses at the early inflammatory stage. However, the administration of microcystin-LR improved the weight recovery, especially if the treatment was initiated in earlier time points (Fig. 1b). Bleomycin-treated rats showed dense deposition of collagen with destruction of normal tissue architecture and a relative increase in the number of inflammatory cells revealed by histological staining. Interestingly, administration of microcystin-LR suppressed bleomycin-induced inflammation and collagen deposition (Fig. 1c-e). The therapeutic effect of microcystin-LR on pulmonary fibrosis was also confirmed in a fluorescein isothiocyanate (FITC)-induced mouse model (Supplementary Fig. S1).

### Microcystin-LR suppresses the TGF-β signaling pathway and reduces the expression levels of fibrotic markers in pulmonary tissues of the model rats

**Fig. 2:**
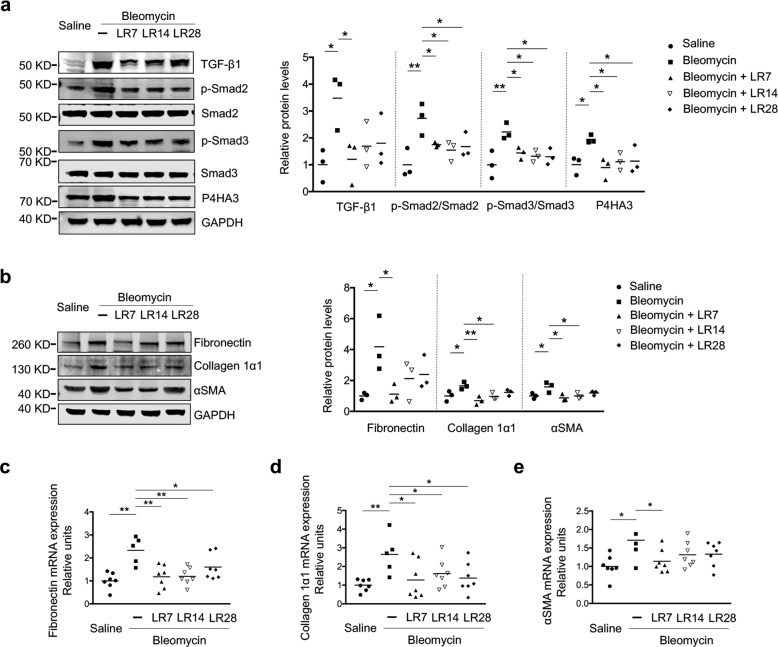
Microcystin-LR antagonizes activation of the TGF-β signaling pathway and expression of fibrotic markers in rats with bleomycin-induced pulmonary fibrosis. Rats were treated as explained in Fig. 1. **a** Expression of TGF-β signaling pathway molecules, including TGF-β1, p-Smad2, Smad2, p-Smad3, Smad3 and prolyl 4-hydroxylase subunit αIII (P4HA3) in rat pulmonary tissues was measured by western blot (one-way ANOVA; TGF-β1: *F*_4,10_ = 3.726, *P* = 0.042; p-Smad2: *F*_4,10_ = 5.790, *P* = 0.011; p-Smad3: *F*_4,10_ = 5.720, *P* = 0.012; P4HA3: *F*_4,10_ = 3.861, *P* = 0.038; *n* = 3). **b** Protein levels of the fibrotic markers, including fibronectin, collagen 1α1 and α-smooth muscle actin (αSMA) in rat pulmonary tissues was measured by western blot (one-way ANOVA; fibronectin: *F*_4,10_ = 3.742, *P* = 0.041; collagen 1α1: *F*_4,10_ = 5.808, *P* = 0.011; αSMA: *F*_4,10_ = 4.722, *P* = 0.021; *n* = 3). **c**–**e** Expression of fibronectin, collagen 1α1 and αSMA at the mRNA level in rat pulmonary tissues was examined by quantitative RT-PCR (qRT-PCR). One-way ANOVA, fibronectin: *F*_4,28_ = 7.024, *P* < 0.000; collagen 1α1: *F*_4,28_ = 3.317, *P* = 0.024; αSMA: *F*_4,28_ = 2.841, *P* = 0.043. *n* = 5 (bleomycin) or *n* = 7 (all the other treatments). *P < 0.05, **P < 0.01 determined by one-way ANOVA with S-N-K multiple-comparison test.

TGF-β1 is the most abundant isoform of TGF-β and has been well established as a key pro-fibrotic mediator in fibrotic diseases^13,23^. As expected, we observed an elevated expression of TGF-β1 in the tissues of bleomycin-induced pulmonary fibrosis (Fig. 2a, Supplementary Fig. S2). Reasonably, we also observed increased phosphorylation levels of Smad2 and Smad3, a measure of TGF-β signaling activity, and upregulation of the prolyl 4-hydroxylase subunit αIII (P4HA3), a novel TGF-β1 downstream target gene, in fibrotic animals^24^. Consistently, the administration of microcystin-LR could suppress the expression of TGF-β1 and reduce activation of the TGF-β/Smad signaling pathway (Fig. 2a, Supplementary Fig. S2). In addition, bleomycin-induced expression of fibrotic markers, including fibronectin, collagen 1α1 and αSMA in rat pulmonary tissues, was significantly reduced following microcystin-LR treatment at most of the observation points (Fig. 2b-e, Supplementary Fig. S2).

### Residual accumulation of microcystin-LR in pulmonary tissues after treatment does not compromise protein phosphatase activity

Microcystin-LR has been characterized as a specific inhibitor of protein phosphatase, mainly protein phosphatase 2A (PP2A) that has been demonstrated to be involved in the regulation of most metabolic pathways and the control of cell cycling^25^. To determine whether the treatment with microcystin-LR could exert anti-fibrotic activities through inhibition of PP2A, we measured the PP2A activity in rat pulmonary tissues. Unexpectedly, we failed to observe any differences in PP2A activity among various treatments (Supplementary Fig. S3a). The liver is regarded as the specific target organ of microcystin-LR. Consistently, we revealed a substantially lower microcystin-LR accumulation in the lungs than in the liver (Supplementary Fig. S3b). Next, we demonstrated that this residual amount of microcystin-LR in rat pulmonary tissues had no inhibition of PP2A activity and retardation of cell yield in vitro (Supplementary Fig. S3c, d).

### The blocking effect of microcystin-LR on EMT and FMT is mediated by monocyte/macrophages

**Fig. 3:**
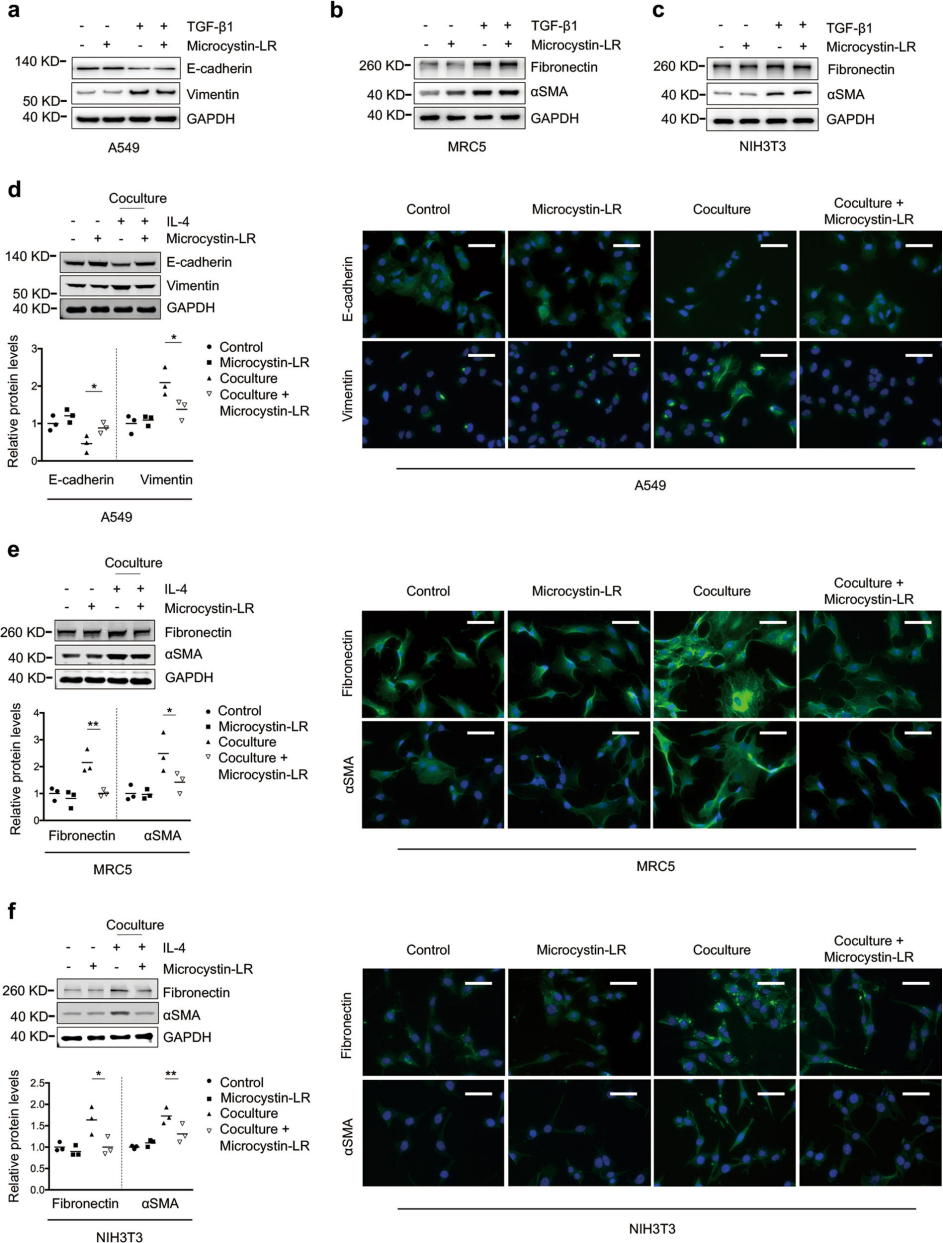
Microcystin-LR targets macrophages to suppress EMT and FMT. **a**–**c** A549 (commonly used as a model of human alveolar type II pulmonary epithelium), MRC5 (human fetal lung fibroblasts) and NIH3T3 (mouse embryonic fibroblasts) were left alone or cultured for 48 h with TGF-β1 (5 ng/ml) to induce epithelial–mesenchymal transition (EMT) or fibroblast-to-myofibroblast transition (FMT). Some of the cells were also treated with 0.1 μM microcystin-LR as indicated. Protein expression in A549 (**a**), MRC5 (**b**) and NIH3T3 (**c**) were examined by western blot. **d**–**f** RAW264.7 cells (murine monocyte/macrophage cell line) were cultured for 48 h with IL-4 (5 ng/ml) to induce the polarization of macrophages in 6-well Millicell hanging cell culture inserts with a membrane pore size of 1.0 μm. Some of the cells were also treated with 0.1 μM microcystin-LR as indicated. Next, cell culture inserts containing the pretreated macrophages were transferred to the plates that were seeded with A549, MRC5 or NIH3T3 cells 24 h ago. After the cell coculture for 48 h, the expression levels of various proteins as indicated in A549 (**d**), MRC5 (**e**) and NIH3T3 (**f**) were examined by western blot (left panel) and immunofluorescence staining (right panel). Densitometric intensity of the bands was quantified (lower panel). *P < 0.05, **P < 0.01 determined by one-way ANOVA with S-N-K post hoc analysis (A549 E-cadherin: *F*_3,8_ = 13.624, *P* = 0.002, Vimentin: *F*_3,8_ = 8.323, *P* = 0.008; MRC5 fibronectin: *F*_3,8_ = 12.317, *P* = 0.002, αSMA: *F*_3,8_ = 7.736, *P* = 0.009; NIH3T3 fibronectin: *F*_3,8_ = 7.681, *P* = 0.01, _α_SMA: *F*_3,8_ = 13.681, *P* = 0.002). Scale bar: 100 μm.

To investigate the effect of microcystin-LR treatment on EMT and FMT responses, we cultured A549 (commonly used as a model of human alveolar type II pulmonary epithelium), MRC5 (human fetal lung fibroblasts) and NIH3T3 (mouse embryonic fibroblast cells) in the presence of TGF-β1 to establish EMT or FMT in vitro. Microcystin-LR did not modulate TGF-β1-induced acquisition of mesenchymal characteristics or fibrotic markers in A549, MRC5 and NIH3T3 cells (Fig. 3a-c). Interestingly, a microcystin-LR-associated inhibition of EMT or FMT was observed when A549, MRC5 or NIH3T3 was cocultured with the murine monocyte/macrophage cell line RAW264.7 that had been pretreated with IL-4 for inducing polarization (Fig. 3d-f). The effect of microcystin-LR treatment on inhibition of EMT or FMT was confirmed with the coculture system of IL-4-induced M2 polarization from bone marrow-derived macrophages (BMDM) and A549, MRC5 or NIH3T3 cells (Supplementary Fig. S4a). However, no significant inhibitory effect of microcystin-LR was observed on EMT and FMT of these epithelial or fibroblast cells when cocultured with M0 or LPS-induced M1 polarization of RAW264.7 (Supplementary Fig. S4b). Additionally, we observed efficient uptake of microcystin-LR in RAW264.7, but minimal uptake in MRC5 and hardly any in A549 cells (Supplementary Fig. S5).

### Microcystin-LR inhibits bleomycin-induced M2 polarization of macrophages in pulmonary tissues

**Fig. 4:**
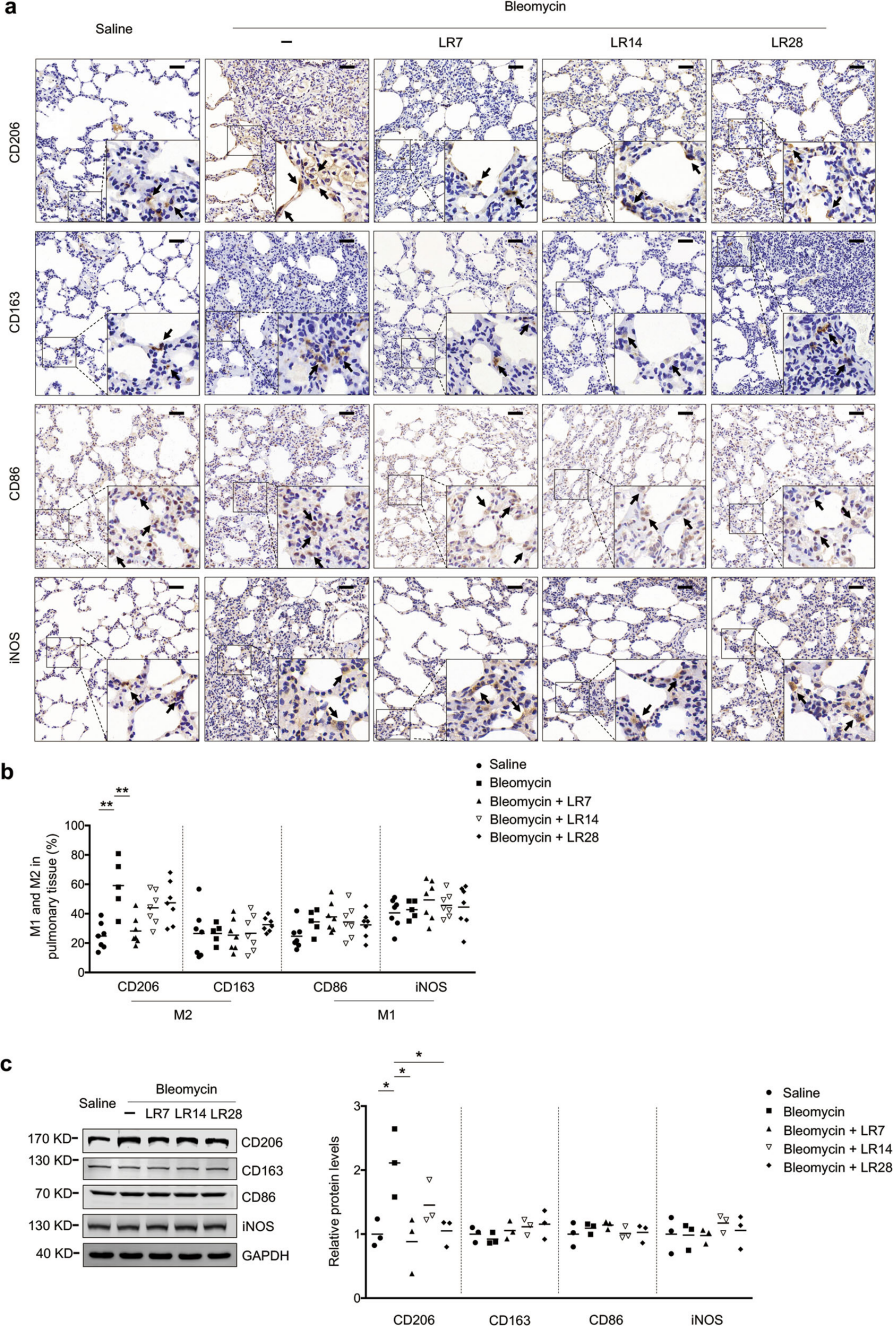
Microcystin-LR alters bleomycin-induced macrophage polarization. Rats were treated as explained in Fig. 1. **a** Pulmonary tissue sections were examined for the expression of CD206 and CD163, markers of alternative activated macrophages, and CD86 and inducible nitric oxide synthase (iNOS), markers as classically activated macrophages using immunohistochemistry. Black arrows indicate CD206, CD163, CD86 or iNOS expression in macrophages. Scale bars: 100 μm. **b** Quantification of M1 and M2 macrophages in rat pulmonary tissues. **P < 0.01 determined by one-way ANOVA with S-N-K post hoc analysis (CD206: *F*_4,28_ = 7.867, *P* = 0.000; CD163: *F*_4,28_ = 0.4_4_1, *P* = 0.778; CD86: *F*_4,28_ = 1.865, *P* = 0.144; iNOS: *F*_4,28_ = 0.618, *P* = 0.653). **c** Total protein was extracted from pulmonary tissues and examined for the expression of CD206, CD163, CD86 and iNOS by western blot. Densitometric intensity of the bands was quantified (right panel). *P < 0.05 determined by one-way ANOVA with S-N-K post hoc analysis (CD206: *F*_4,10_ = 5.493, *P* = 0.013_;_ CD163: *F*_4,10_ = 1.181, *P* = 0.376_;_ CD86: *F*_4,10_ = 0.725, *P* = 0.59_4;_ iNOS: *F*_4,10_ = 0.437, *P* = 0.779).

Immunohistochemistry staining was performed to make an investigation on whether treatment with microcystin-LR could alter bleomycin-induced macrophage responses. The results showed that microcystin-LR inhibited the bleomycin-induced CD206^+^ macrophage differentiation but failed to impact on CD163^+^ macrophages in rat pulmonary tissues (Fig. 4). Although both CD206 and CD163 represent M2 markers, CD206 is positively expressed on M2a subpopulations, mainly responsible for anti-inflammatory responses and induction of tissue fibrosis, whereas CD163 on M2c macrophages indicates immunosuppression and tissue repair. However, microcystin-LR treatment did not exert significant impact on the expression of CD86 and inducible nitric oxide synthase (iNOS), two markers of M1 macrophages (Fig. 4). On the other hand, the cells with CD68^+^, a pan marker for macrophage, were significantly increased in the lung tissues of bleomycin-induced model rats, comparing with saline controls. But there is no statistical difference on CD68^+^ macrophages among the bleomycin-induced rats with or without microcystin-LR treatment (Supplementary Fig. S6). Furthermore, microcystin-LR was observed to counteract, at the mRNA level, bleomycin-induced upregulation of anti-inflammatory molecules of M2 macrophages. However, microcystin-LR used in model animals did not significantly alter the mRNA expression of pro-inflammatory molecules characterized by M1 macrophages in the pulmonary tissues (Supplementary Fig. S7). These results suggest that microcystin-LR could reduce the numbers of CD206 + M2-like macrophages. The amelioration of bleomycin-induced pulmonary fibrosis by microcystin-LR treatment may be associated with an alteration of the macrophage polarization.

### Microcystin-LR modulates macrophage polarization by suppressing GRP78-mediated responses to endoplasmic reticulum stress

**Fig. 5:**
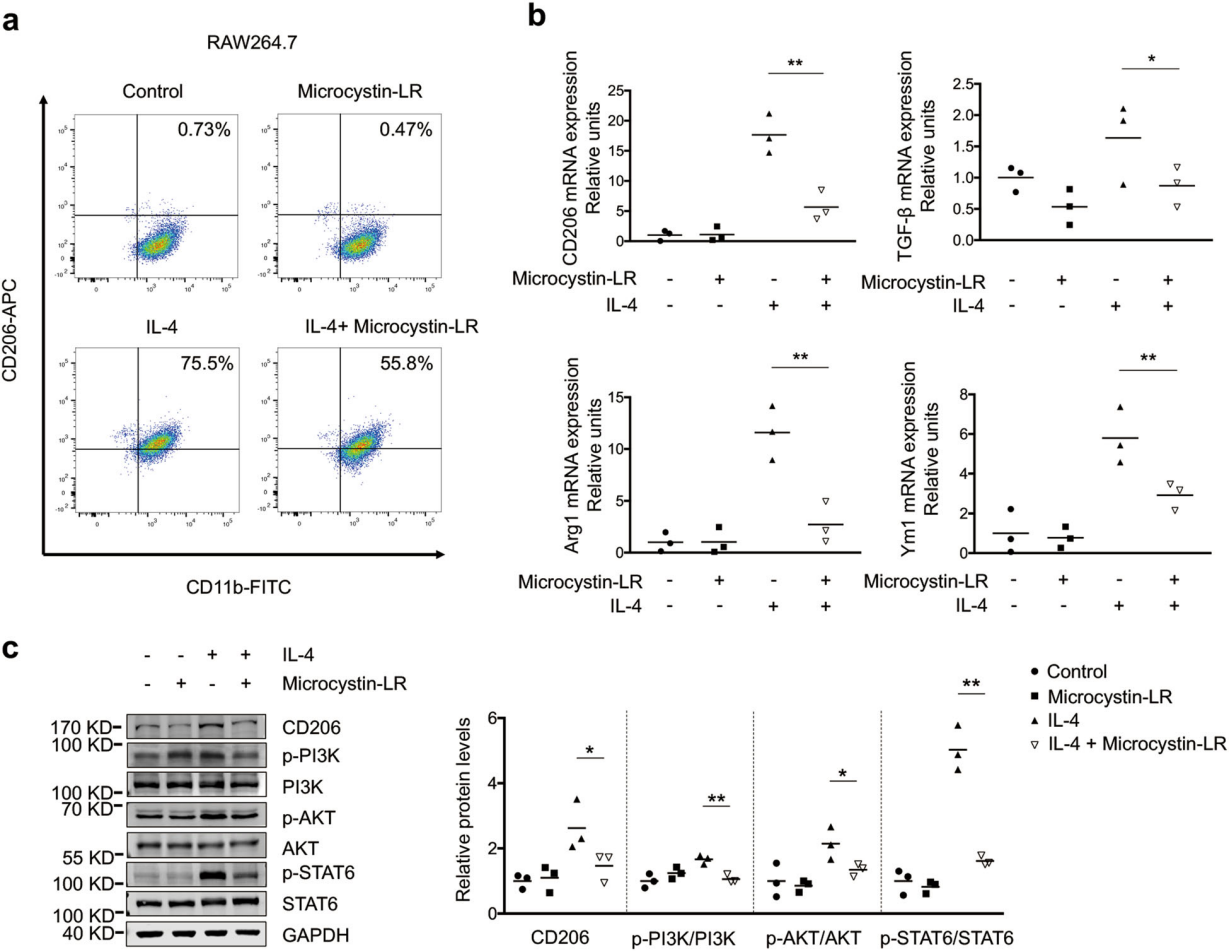
Microcystin-LR has inhibitory effect on IL-4 induced M2 polarization of macrophages. RAW264.7 cells were left alone or treated with IL-4 (5 ng/ml) for 48 h to induce polarization. In some of the assays, cells were also treated with 0.1 μM microcystin-LR for the same duration as indicated. **a** Percentages of CD206^+^/CD11b^+^ M2-like macrophages were analyzed by flow cytometry. **b** Relative expression of the M2-like macrophage markers CD206, TGF-β1, arginase-1 (Arg1) and chitinase-like 3 (Ym1) was analyzed using qRT-PCR. *P < 0.05, **P < 0.01 determined by one-way ANOVA with S-N-K post hoc analysis (CD206: *F*_3,8_ = 38.234, *P* < 0.000_;_ TGF-β1: *F*_3,8_ = 6.900, *P* = 0.013; Arg1: *F*_3,8_ = 23.223, *P* < 0.000; Ym1: *F*_3,8_ = 15.990, *P* = 0.001; *n* = 3). **c** Protein levels of CD206, p-PI3K/PI3K, p-AKT/AKT and p-STAT6/STAT6 signaling pathway molecules were determined by western blot. *P < 0.05, **P < 0.01 determined by one-way ANOVA with S-N-K post hoc analysis (CD206: *F*_3,8_ = 6.475, *P* = 0.016; PI3K: *F*_3,8_ = 9.546, *P* = 0.005; AKT: *F*_3,8_ = 6.813, *P* = 0.014; STAT6: *F*_3,8_ = 67.900, *P* < 0.000; *n* = 3).

To explore the mechanisms of microcystin-LR-mediated macrophage polarization, we set up an IL-4-dependent monocyte/macrophage (RAW264.7) activation model. Microcystin-LR was found to be capable of reducing the portion of CD206^+^ pool in RAW264.7 cells by flow cytometry (Fig. 5a). Next, we confirmed microcystin-LR-mediated down-regulation of the transcriptional expression of CD206, TGF-β1, arginase-1 (Arg1) and chitinase-like 3 (Ym1) (Fig. 5b). Furthermore, treatment with microcystin-LR could inhibit the level of p-PI3K, p-AKT and p-STAT6 as evidenced by antagonizing IL4-induced polarization to M2-like monocyte/macrophages (Fig. 5c)^26,27^. Moreover, it is failed to observe any discernible signs of regulation on cell cycling and apoptosis of RAW264.7 with microcystin-LR treatment (Supplementary Fig. S8).

**Fig. 6:**
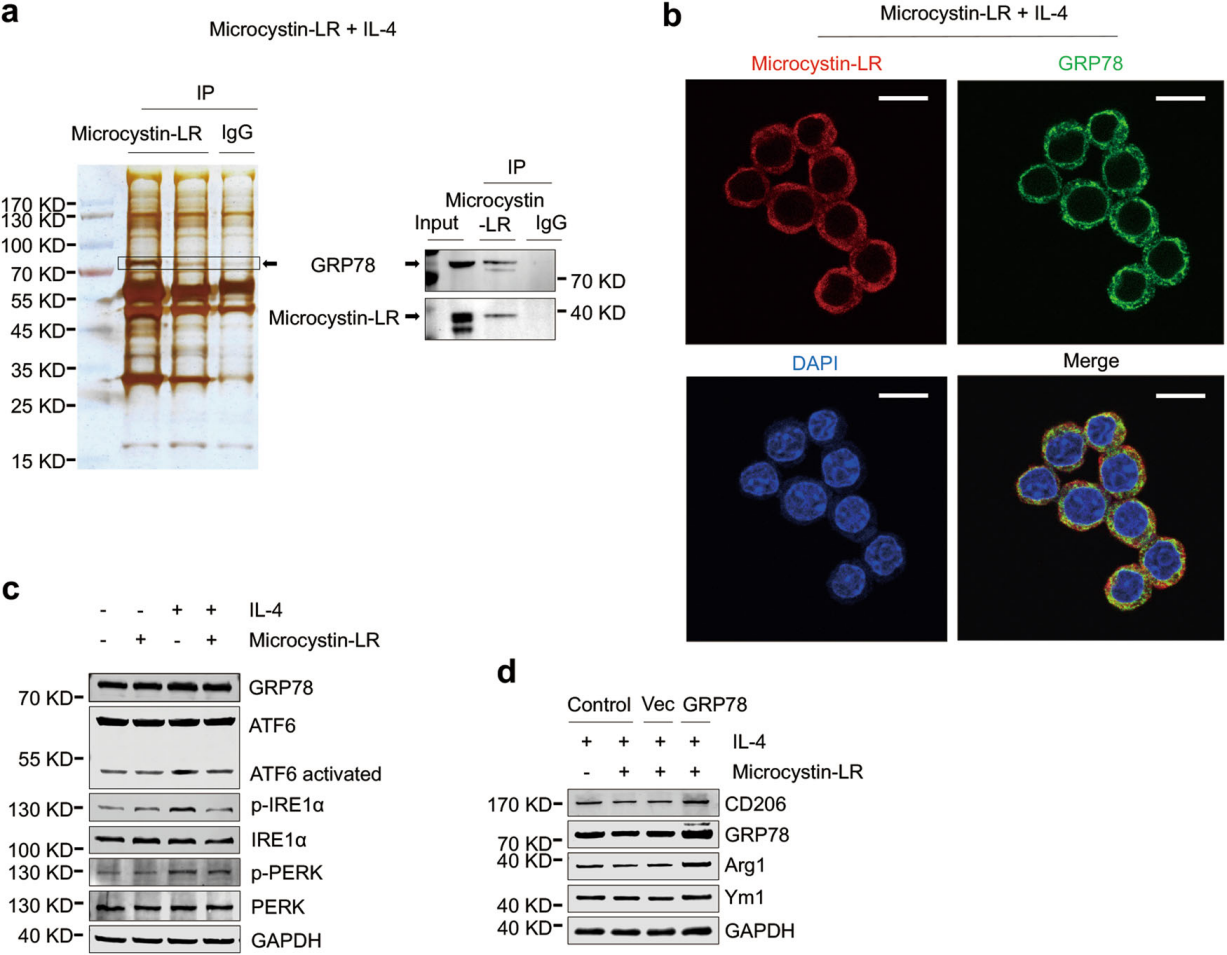
Microcystin-LR inhibits the responses to endoplasmic reticulum stress by interaction with GRP78. RAW264.7 cells were treated as explained in Fig. 5. **a** Total protein extracts from RAW264.7 cells pretreated with IL-4 and microcystin-LR were immunoprecipitated with two independent microcystin-LR antibodies (left lane: MOB-647, Creative Biolabs; middle lane: ALX-804-320, Alexis Biochemicals). A ~ 78 kDa protein band was subsequently identified as glucose-regulated protein 78 kDa (GRP78) using mass spectrometry (left panel) and western blot (right panel). **b** Subcellular localization of microcystin-LR (red) and GRP78 (green) was examined using immunofluorescence staining. DAPI was used for nuclear staining (blue). Scale bar: 50 μm. **c** Cellular protein expression of GRP78 and its three transmembrane sensors including activating transcription factor 6 (ATF6), inositol requiring enzyme 1α (IRE1α) and PKR-like endoplasmic reticulum kinase (PERK), together with their activated forms as indicated was measured by western blot. **d** GRP78 rescue assay was performed by transfecting RAW264.7 cells with the recombinant GRP78 expression plasmid (pcDNA3-GRP78). Control cells without transfection, vector (pcDNA3.1) and the pcDNA3-GRP78 transfected cells were treated as indicated for 48 h. The expressions of M2 macrophage markers, CD206, Arg1 and Ym1, and GRP78 were analyzed by western blot.

With immunoprecipitation and protein mass spectrometry techniques, we further identified that microcystin-LR has an interaction with glucose-regulated protein 78 (GRP78), a chaperonin and a master regulator of the endoplasmic reticulum (ER) unfolded protein response (UPR^ER^) in ER stress (Fig. 6a). We next observed a strong overlap of GRP78 and microcystin-LR for the subcellular localization in IL-4-induced RAW264.7 cells (Fig. 6b). ER stress facilitates macrophage acquisition of the M2-like phenotype. As shown in Fig. 6c, microcystin-LR modulated ER stress in IL-4-induced RAW264.7 by reducing the levels of the three activated UPR^ER^ signaling sensors, phosphorylated PKR-like endoplasmic reticulum kinase (p-PERK), phosphorylated inositol requiring enzyme 1α (p-IRE1α) and cleaved activating transcription factor 6 (ATF6 activated)^28,29^. Additionally, our data also revealed that GRP78 overexpression could reverse microcystin-LR-mediated inhibition of CD206^+^ M2-like phenotype (Fig. 6d). Next, immunofluorescence staining confirmed the localization of GRP78 with CD206 was overlapping in the pulmonary resident cells. As expected, GRP78 was abundantly expressed in CD206^+^ cells, but the numbers of double-labeled cells were reduced in all the microcystin-LR treatment rats (Supplementary Fig. S9). These results indicated that the interaction of microcystin-LR with GRP78 might have a key regulatory role in the alleviation of ER stress and in the modulation of CD206^+^ monocyte/macrophage differentiation.

### The regimen of microcystin-LR does not cause functional damage in bleomycin-instilled animals

To investigate whether the regimen of microcystin-LR used in this study caused extra damage to rats with bleomycin-induced pulmonary fibrosis, we performed blood tests and histological examination. Our data exhibited no observable adverse effect on serum biochemistry with microcystin-LR treatment (Supplementary Table 1). The blood cell counting indicated that microcystin-LR could suppress bleomycin-associated increases in neutrophils and monocytes (Supplementary Fig. S10). Histological examination of liver and kidneys, two susceptible organs to microcystin exposure, failed to identify any tissue damage in the rats with microcystin-LR treatment (Supplementary Fig. S11). Based on these data, it was believed that the regimen of microcystin-LR for developing a therapeutic strategy against pulmonary fibrosis would not be accompanied with substantial toxic damage.

## Discussion

We conceived this study based on our previous observation of a reduced expression of TGF-β1 in mouse lung tissues after chronic exposure to microcystin-LR. Given that TGF-β1 is one of the most important profibrotic cytokines, we hypothesized that microcystin-LR could possibly have an effect on pulmonary fibrosis. In the previous work, mice received microcystin-LR in drinking water at five concentrations (1, 5, 10, 20, and 40 μg/L) for twelve months^22^. Decreased expression of TGF-β1 appeared in the mice exposed to microcystin-LR equal to or above 20 μg/L in drinking water (data not published). Therefore, 20 μg/L was selected as the dose of microcystin-LR in drinking water in our current research.

Bleomycin-induced pulmonary fibrosis is the most commonly used model of fibrosis in rodents, and it manifests similar characteristics of IPF^30,31^. Most previous works that focused on a beneficial antifibrotic substance or molecule for bleomycin-induced pulmonary fibrosis was usually carried out in a preventive regimen, with the drug given within 7 days after bleomycin administration^32^. In this work, we have provided compelling evidence demonstrating that microcystin-LR has a therapeutic effect on the rats with bleomycin-induced pulmonary fibrosis, especially when microcystin-LR was administered during early or inflammation/fibrosis transitional phase of the fibrotic development. To confirm the therapeutic effect of microcystin-LR on pulmonary fibrosis independent of bleomycin exposure, we treated FITC-induced pulmonary fibrosis with microcystin-LR in mice and observed a similar remission of fibrosis.

Myofibroblasts, originated from exaggerated activation and abnormal differentiation of fibroblasts via EMT and FMT pathways, are widely considered as the effector cells responsible for fibrosis^33,34^. A prolonged inflammation, M2 macrophages differentiation and increased profibrotic factors, especially TGF-β1/Smad signaling form and maintain the microenvironment of fibrogenesis. Therapeutic approaches either by repressing TGF-β1/Smad signaling or via targeting M2 macrophages could generate a potential effect on alleviating pulmonary fibrosis^35,36^. Using a cell coculture system, A549, MRC5 and NIH3T3 cells cocultured with IL-4-induced macrophages (RAW264.7 or mouse BMDM), we revealed that microcystin-LR blocked the EMT or FMT pathways through modulating M2 polarization of macrophages, rather than directly affecting epithelial cells or the fibroblast phenotype. Given the number of CD206 + macrophages, but not CD163^+^, CD68^+^, CD86^+^ or iNOS^+^ macrophages, was markedly decreased upon microcystin-LR treatment, it was reasonable to assume that microcystin-LR could meliorate pulmonary fibrosis mainly by suppressing CD206^+^ M2a-like macrophage differentiation. Human-derived macrophage polarization will be used in the coculture system in our future work although TGF-β1 shows a cross-species activity.

We next attempted to clarify the issues involved in microcystin-LR-mediated macrophage differentiation. Using immunoprecipitation with two independent microcystin-LR antibodies and protein mass spectrometry, we unexpectedly identified that microcystin-LR was capable of interacting with GRP78, a chaperonin associated with ER stress. This result was confirmed by western blot. Knarr et al. developed a computer program to predict GRP78 (also known as BiP)-binding sites in proteins by scoring amino acid sequences based on the segments of seven residues, and then test them with synthetic heptapeptides corresponding to the potential binding sites. They confirmed that GRP78 is associated with a wide variety of target proteins and the binding peptides for GRP78 are enriched in hydrophobic residues. Microcystin-LR is a cyclic heptapeptides with hydrophobic (2S, 3S, 8S, 9S)-3-amino-9-methoxy-2-6-8-trimethyl 10-phenyldeca-4,6-dienoic acid (Adda) side chains, which allows interaction with GRP78^37,38^. Chun et al. reported that the holoenzyme of PP1γ2, a testis-specific isotype of protein phosphatase 1, is a heterotrimer-containing GRP78 subunit. Removing GRP78 from the heterotrimer of PP1γ2 results in a significant loss of enzymatic activity and renders PP1γ2 less sensitive to its inhibitor microcystin-LR^39^. Both the mentioned works also support a potential interaction between GRP78 and microcystin-LR, which we demonstrated in the current study. Increasing evidence suggests that the ER stress controls M2 phenotype differentiation of macrophages^40,41^. Under ER stress, GRP78 frees its client proteins, which leads to phosphorylation of PERK and IRE1α, and cleavage of ATF6, and contributes to the initiation of the UPR^ER^ signaling pathway. Our further investigation demonstrated that microcystin-LR could suppress the UPR^ER^ signaling pathway through downregulating p-PERK, p-IRE1α and the cleaved ATF6. Overexpression of GRP78 antagonized microcystin-LR-mediated inhibition of CD206^+^ macrophage polarization, suggesting microcystin-LR could avert activation of GRP78. Kaufman and Schroder reported a dynamic equilibrium between monomeric and oligomeric GRP78 in cells^42^. In the oligomeric status, the peptide-binding domain of GRP78 is in phosphorylation and forms a protein complex with PERK, IRE1α and ATF6. In ER stress, the oligomeric GRP78 can dissociate into dephosphorylated monomer that binds to the unfolded proteins, releasing three ER transmembrane sensors to activate downstream signaling pathway molecules. Thus, we speculate that the physical binding between microcystin-LR and GRP78 may prevent the GRP78 protein from dephosphorylation catalyzed by protein phosphatases, which will in turn impede the regulation of GRP78 on UPR^ER^ signaling pathway.

Microcystin-LR is a well characterized environmental toxicant absorbed by human body via the oral route and accumulates in the liver, kidney, lung and other tissues. It was compulsory to assess whether the regimen of microcystin-LR in the current study may cause additional lesion in the rats of bleomycin-induced pulmonary fibrosis. Fawell et al. implemented several sets of experiments to investigate the toxicity of microcystin-LR in rodents. They demonstrated that the mice exposed to microcystin-LR with the dose of 40 μg/kg body weight per day by gavage for 13 weeks have no observed adverse effect level (NOAEL) for liver tissues^43^. In this study, we treated rats with drinking water containing 20 μg/L microcystin-LR, which is equivalent to 3 μg/kg/day intake (calculated based on daily water consumption of 1.5 ml/10 g body mass) for 7, 6 and 4 weeks, respectively. We previously described the damage of liver tissue in the mice following chronic exposure to microcystin-LR for twelve months^22^. The duration of microcystin-LR treatment used in the current study was much shorter compared with our previous experiments. Therefore, it is not surprising that we failed to identify any discernable toxic effects of microcystin-LR on the model rats. Further investigations should be performed to identify the structural analogues with the same efficacy but lower toxicity in microcystins for the treatment of pulmonary fibrosis.

**Fig. 7:**
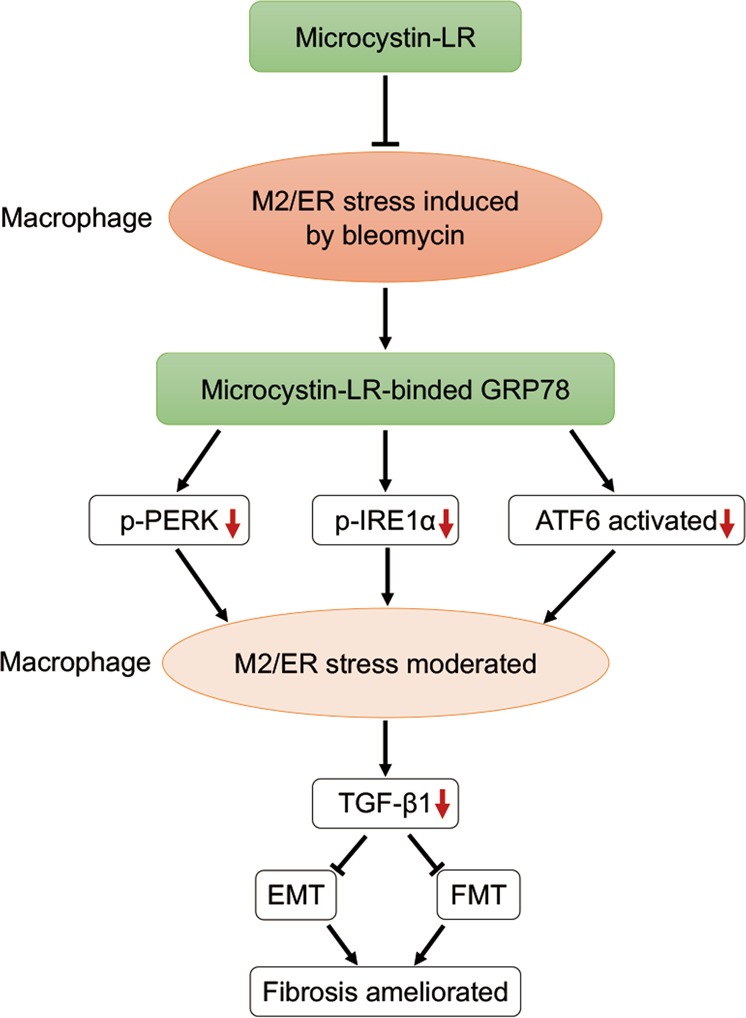
Model of the proposed mechanism by which microcystin-LR ameliorates pulmonary fibrosis. Microcystin-LR is selectively taken into macrophages in lung tissue and binds to GRP78, a master regulator of ER stress, resulting in attenuation of M2 polarization of macrophages through reduction of p-PERK, p-IRE1α and cleaved ATF6. The macrophages with modulated ER stress decrease the expression of TGF-β1, which causes a repressed epithelial–mesenchymal transition and fibroblast–myofibroblast transition, and leads to the amelioration of pulmonary fibrosis.

Microcystin-LR can attenuate macrophage polarization toward a CD206^+^ M2-like phenotype, resulting in the inhibition of EMT or FMT signaling in epithelial cells and fibroblasts. Furthermore, we demonstrated an interaction between microcystin-LR and GRP78, a master regulator of ER stress, which led to a blockage effect on UPR^ER^ signal transduction in the stressed cells. This may well be a possible mechanism underlying microcystin-LR-mediated attenuation of CD206^+^ macrophage differentiation and reduction of TGF-β1 expression, which would result in amelioration of pulmonary fibrosis (Fig. 7). Our results provide a novel insight on the therapeutic exploitation of microcystin-LR, an enigmatic natural molecule classically described as a cytotoxic agent, for the treatment of IPF.

## Materials and methods

### Chemicals

Microcystin-LR was purchased from Alexis Biochemicals (Lausen, Switzerland), while bleomycin was purchased from Nippon Kayaku Co. (Tokyo, Japan). Human TGF-β1, murine macrophage colony-stimulating factor (M-CSF) and IL-4 were purchased from Pepro Tech (Rocky Hill, New Jersey, USA). FITC and LPS was purchased from Sigma-Aldrich (St. Louis, Missouri, USA).

### Rat model

Six-eight weeks old male SD rats (220–250 g in weight) were provided by the Model Animal Research Center of Nanjing University (Nanjing, China). The animals were housed in a special pathogen-free, and temperature- and humidity-controlled (23 °C ± 2 °C and 50% relative humidity) room with a 12-h light/dark cycle. Water and rodent chow were freely available. All of the rats were acclimated for 7 days and then completely randomized into five groups. For the induction of pulmonary fibrosis, rats were intratracheally instilled with a single dose of bleomycin (5.0 mg/kg) on day 0. Rats that received intratracheal instillation of an equal volume of saline were used as control. In some of the rats with fibrosis induction, the animals received microcystin-LR (20 μg/L) in drinking water in three groups, starting on day 7 (LR7), 14 (LR14) or 28 (LR28), respectively. All rats were euthanized 56 days after receiving bleomycin intratracheal instillation for further analysis (Fig. 1a).

### Mouse model

Six–eight weeks old male C57BL/6 mice (20–22 g) were purchased from Model Animal Research Center of Nanjing University. The feeding environment and conditions of mice were the same as described in the rat model. Mice were completely randomized into three groups. Some mice were intratracheally instilled with a single dose of FITC (3.5 mg/kg) on day 0 to induce pulmonary fibrosis. Saline was administered similarly as a control. For microcystin-LR treatment, the mice with fibrosis induction received microcystin-LR (20 μg/L) in drinking water starting on day 14. All animals were euthanized on day 56 (Supplementary Fig. S1a). Tissue samples were collected followed by fixation with a 10% neutralized formalin solution for 24 h at room temperature and the remaining tissues were kept at −80 °C.

### Hydroxyproline assay

The collagen content in lung homogenates was evaluated by a hydroxyproline (HYP) assay using a kit from Nanjing JianCheng Bioengineering Institute (Nanjing, China). The HYP assay was conducted essentially according to the manufacturer’s instruction. The absorbance of each sample was read at 550 nm in a SpectraMax M2e microplate reader (Molecular Devices, Sunnyvale, California, USA). The experiment was performed in triplicate and data presented as μg/mg protein of the lung tissues.

### PP2A phosphatase activity assay

PP2A activity was determined by a serine/threonine phosphatase assay kit in accordance with the manufacturer’s protocols (Promega, Madison, Wisconsin, USA). The total proteins of the lung tissues or the cultured L02 cells were extracted in a phosphatase lysis buffer (50 mM Tris-HCl pH 7.5, 10% glycerol, 0.05% β-mercaptoethanol, 0.1 mM EDTA, 0.05% Triton X-100, 0.5 mM PMSF, phosphatase inhibitor cocktail) and measured for phosphatase activity using a PP2A-type specific buffer (250 mM imidazole pH 7.2, 1 mM EGTA, 0.1% β-mercaptoethanol, 0.5 mg/ml bovine serum albumin). Free phosphate, generated from a synthetic phosphothreonine peptide RRA(pT)VA-specific for PP2A, was quantified by measuring the molybdate/malachite green/phosphate complex at 630 nm.

### Quantification of microcystin-LR in the lung and liver tissues

The retention of microcystin-LR in the lung and liver tissues was assessed by an ELISA kit (Alexis Biochemicals, Lausen, Switzerland). Briefly, tissue samples were homogenized directly in a lysis buffer for protein extraction and diluted ten-fold to fall within the microcystin-LR detection range according to the manufacturer’s instructions. Plates were read at 550 nm.

### Histology and immunostaining

For histologic evaluation, the fixed right lung tissues were embedded in paraffin, sectioned (4 μm) onto glass slides and stained with H&E for structured observation and Masson’s trichrome for detection of collagen deposits. The development of pulmonary lesions was scored by two pathologists blind to the study design. For immunohistochemistry staining, the sections were permeabilized in 1× PBS containing Triton X-100 (0.1%) for 10 minutes and then probed with antibodies against CD206 (ab64693, Abcam, Cambridge, Massachusetts, USA), CD163 (ab182422, Abcam), CD86 (BS9900M, Bioworld, St. Louis Park, Minnesota, USA), iNOS (ab178945, Abcam), CD68 (ab213363, Abcam), TGF-β1 (ab215715, Abcam) and αSMA (ab32575). For the immunofluorescence assay, slides were first labeled with a mouse-derived antibody against CD206 (ab8918, Abcam) and a rabbit-originated GRP78 (ab21685, Abcam) antibody followed by staining with an Alexa Fluor 595-labelled anti-mouse and Alexa Fluor 488-conjugated anti-rabbit antibodies (Thermo Fisher Scientific, Inc., Waltham, Massachusetts, USA). Images were captured using a Zeiss Axio upright fluorescent microscope.

### Cell culture

Cell lines including L02, A549, MRC5, NIH3T3 and RAW264.7 were obtained from Cell Bank of Typical Culture Preservation Commission, Chinese academy of Sciences. Three human-derived cell lines (L02, A549 and MRC5) were authenticated by STR profiling. All cells were tested negative for mycoplasma contamination using MycoBlue^TM^ mycoplasma detector (Vazyme, Nanjing, China). L02, NIH3T3 and RAW264.7 cells were cultured in DMEM medium (Thermo Fisher Scientific). A549 was maintained in RPMI 1640 medium (Thermo Fisher Scientific) and MRC5 in low-glucose DMEM medium (Thermo Fisher Scientific). Bone marrow mononuclear cells from C57BL/6 mice were isolated and treated with M-CSF (20 ng/ml) in DMEM medium for differentiation into macrophages prior to experiments. All cell media were supplemented with 10% fetal bovine serum (Thermo Fisher Scientific). Cells were cultured at 37 °C in a 5% CO_2_ humidified incubator to reach 80% of confluence.

### Treatment of the cultured cells and coculture

A549, MRC5 or NIH3T3 cells with a seeding density of 1×10^5^ cells/ml in six-well plates were cultured in medium containing TGF-β1 (5 ng/ml) for 48 h to induce EMT or FMT in vitro. Microcystin-LR (0.1 μM) was synchronously used to observe the intervention effect on the treated cells. RAW264.7 cells or mouse BMDM seeded at a density of 1×10^5^ cells/ml were exposed to LPS (10 ng/ml) for 48 h to induce M1- or exposure to IL-4 (5 ng/ml) for 48 h to induce M2-like differentiation. Some of the cells were also treated with 0.1 μM microcystin-LR. To establish the coculture with M1 or M2-like macrophages, we transferred the cell culture inserts containing LPS or IL-4 pretreated macrophages (as mentioned above) to the plates that had been seeded with A549, MRC5 or NIH3T3 cells (5×10^4^ cells/ml) for culturing 24 h. After 48 h of coculture, the cells at the bottom of the plates were harvested for further experiments.

### Cell proliferation assay

Cell yield was measured in real time using xCELLigence system (ACEA Biosciences Inc., San Diego, California, USA). Firstly, 50 μl medium was added to each well of E-Plate for the impedance baseline measurement. Next, the final volume was adjusted up to 150 μl by the addition of 100 μl culture medium containing 5000 cells in each well. The E-Plate was incubated at 37 °C with 5% CO_2_ for 20 h and then microcystin-LR was added to the wells at the indicated concentrations. The E-Plate continued to be incubated and cell yield was monitored at a 15-minute interval for up to 48 h.

### Flow cytometry

RAW264.7 cells were left alone or cultured with IL-4 (5 ng/ml) for 48 h. Some of the cells were also treated with 0.1 μM microcystin-LR. Cells were harvested and washed twice with 3 ml 0.5% BSA-PBS by centrifuging at 500 g for 5 min at 4 °C. The cell pellet was suspended in 1 ml 0.5% BSA-PBS and the total number was calculated by a cell counter. Following preincubation with mouse Fc block (eBioscience, San Diego, California, USA) for 15 min, the cell suspension was stained with anti-mouse CD11b-FITC (eBioscience) and CD206-APC (eBioscience) at 4 °C for 30 min in the dark. After washing and resuspending in PBS, the cells were analyzed by a flow cytometer (FACSCalibur, BD Biosciences, San Jose, California, USA). For PI and Annexin-V double staining, cells were suspended in 100 μl of a buffer solution (10 mM HEPES/NaOH, 140 mM NaCl, 2.5 mM CaCl_2_, 5 mM KCl, pH 7.4) and stained with FITC-conjugated AnnexinV and PI (50 μg/ml) for 30 min at room temperature in the dark.

### Plasmid recombination and cell transfection

The expression plasmid pcDNA3.1 was purchased from Thermo Fisher Scientific. The recombinant GRP78 expression plasmid (pcDNA-GRP78), containing full-length rat GRP78 cDNA, was constructed. When the cultured cells reached 60–80% of confluence, the expression vectors were transiently transfected into the indicated cells with lipofectamine 3000 (Thermo Fisher Scientific) following the manufacturer’s instructions. The transfected cells were then cultured for 48 h and harvested for analysis.

### Immunoprecipitation and mass spectrometry

RAW264.7 cells were treated with IL-4 (5 ng/ml) and microcystin-LR (0.1 μM) for 48 h followed by harvesting. The cells were lysed using immunoprecipitation lysis/wash buffer (Thermo Fisher Scientific) containing a Halt protease and phosphatase inhibitor cocktail (Thermo Fisher Scientific). The cell extracts left on a rocking platform for 1 h, protein concentration was determined using the pierce BCA protein assay (Thermo Fisher Scientific). Protein from 500 μg to 1 mg was incubated with two clones of microcystin-LR antibodies separately (MOB-647, Creative Biolabs, Shirly, New York, USA and ALX-804–320, Alexis Biochemicals) overnight at 4 °C. The protein A/G PLUS-Agarose beads (Santa Cruz Biotechnology Inc., Santa Cruz, California, USA) were subsequently applied for 2 h at 4 °C. After centrifugation, the beads were collected and washed 5 times with IP Lysis Buffer. The samples were then boiled in 1× SDS solution at 95 °C for 5 min to elute the immunocomplexed proteins. The eluted products were separated in SDS-PAGE, followed by silver staining (Thermo Fisher Scientific) according to the manufacturer’s protocol. Candidate protein bands were carefully cut for mass spectrometry analysis provided by Hoogen Biotech (Shanghai, China).

### Western blot

The cells and tissues were homogenized and lysed in the RIPA buffer supplemented with proteinase inhibitors. Equal amounts of proteins were loaded and separated on a 10% SDS-PAGE gel. Following electrophoresis, the proteins were transferred to a PVDF membrane, blocked in 5% (w/v) non-fat milk and incubated with the primary antibodies overnight. The sources of primary antibodies were: TGF-β1 (ab215715, Abcam), αSMA (ab32575, Abcam), Fibronectin (ab32419, Abcam), Collagen 1α1 (ab138492, Abcam), Smad2/3 (3102, Cell Signaling Technology, Beverly, Massachusetts, USA), p-Smad2 (ab53100, Abcam), p-Smad3 (ab138659, Abcam), P4HA3 (ab101657, Abcam), E-cadherin (3195S, Cell Signaling Technology), Vimentin (5147S, Cell Signaling Technology), CD163 (BS71200, Bioworld), CD206 (ab125028, Abcam), CD86 (BS9900M, Bioworld), iNOS (ab178945, Abcam), PI3K (4249T, Cell Signaling Technology), p-PI3K (4228T, Cell Signaling Technology), AKT (A5031, Selleckchem, Houston, Texas, USA), p-AKT (A5030, Selleckchem), p-STAT6 (56554S, Cell Signaling Technology), STAT6 (5397S, Cell Signaling Technology), microcystin-LR antibody (ALX-804-320, Alexis Biochemicals), GRP78 (ab21685, Abcam), PERK (BS2156, Bioworld), p-PERK (YP1055, ImmunoWay Biotechnology, Planto, Texas, USA), IRE1α (ab37073, Abcam), p-IRE1α (ab226974, Abcam), ATF6 (BS6476, Bioworld), Arg1 (ab60176, Abcam) and Ym1 (ab93034, Abcam). The membranes were washed three times and incubated in a 0.1% tween phosphate buffer solution (PBST) for 10 min followed by probing with goat anti-mouse or goat anti-rabbit IR-Dye 800cw for 1.5 h at room temperature. The signal was detected using an Odyssey scanner (Li-Cor Biosciences, Lincoln, Nebraska, USA).

### Quantitative RT-PCR analysis

Total RNA was isolated from cells and pulmonary tissues using TRIzol reagent (Thermo Fisher Scientific), and qRT-PCR was performed to examine the target RNA using Fast SYBR^®^ Green Master Mix (Thermo Fisher Scientific) on a QuantStudio6 (Applied Biosystems, Foster City, California, USA) as directed by the manufacturer. Each sample was tested in triplicate, and at least two biological samples were included in each assay. Results of mRNA quantification were normalized against GAPDH and calculated using the ΔΔ cycle threshold (Ct) method. Forward and reverse primer sequences for specific genes are listed in Supplementary Table 2.

### Immunofluorescence assay

Cells were fixed with 4% paraformaldehyde (Thermo Fisher Scientific) in PBS, permeabilized with 0.1% Triton X-100 (Thermo Fisher Scientific) in PBS, and then blocked in 3% BSA (Sigma-Aldrich). Subsequently, cells were incubated with specific primary antibodies overnight at 4 °C. The following primary antibodies at indicated dilutions were used: microcystin-LR (Creative Biolabs, 1:50), Vimentin (Cell Signaling Technology, 1:200), E-cadherin (Cell Signaling Technology, 1:200), Fibronectin (Abcam, 1:200), αSMA (Abcam, 1:200), GRP78 (Abcam, 1:200). Cells were stained with Alexa Fluor 488- and Alexa Fluor 549-labeled secondary antibodies (Thermo Fisher Scientific, 1:300) for 1 h at room temperature in the dark. Nuclear staining was performed with DAPI (Thermo Fisher Scientific, 1 μg/ml in PBS) for 10 minutes at room temperature. The images were observed on an Olympus confocal laser scanning microscope imaging system and a Zeiss fluorescent microscope.

### Hematological analysis and serum biochemistry

Blood samples were collected from the rats for measuring the hematological parameters. Blood cells were differentially counted with an Automatic Blood Analyzer (Sysmex, Kobe, Japan). Serum parameters, including AST, ALT, BUN, and CRE, and the lipid profile, were assayed by an Auto-dry Chemistry Analyzer (Kehua Bioengineering, Shanghai, China).

### Statistical evaluation

Statistical analysis was carried out using SPSS 24.0 (SPSS Inc., Chicago, Illinois, USA). Data were expressed using plotting individual value. Normal distribution of the data was tested. All two-sample and multiple-comparison tests were two-tailed. Differences in body weight gain ratios in experimental animals that received various treatments were assessed by repeated measure ANOVA. Statistical significance for multiple comparisons was evaluated by one-way ANOVA with S–N–K post hoc analysis. For comparisons of microcystin-LR concentration in rat liver and lung tissues, data were analyzed using paired Student’s *t* test. *P* < 0.05 was considered statistically significant. The sample size for rat model was calculated using power analysis based our pilot study. Type I error is fixed at the level of 5% (*P* = 0.05). Power is kept at 90%. Supposed sample size was estimated by formula (completely randomized design for multiple means comparison). The sample size for FITC-induced mice pulmonary fibrosis was determined as previously described^44^.

### Study approval

The animal care and the study procedures were approved by the Ethics Committee for Animal Research in Medical School of Nanjing University.

## Supplementary information

Supplementary figure legends

Supplementary table 1

Supplementary table 2

Supplementary figure 1

Supplementary figure 2

Supplementary figure 3

Supplementary figure 4

Supplementary figure 5

Supplementary figure 6

Supplementary figure 7

Supplementary figure 8

Supplementary figure 9

Supplementary figure 10

Supplementary figure 11
