# Supplementary material for: Microcystin-LR ameliorates pulmonary fibrosis via modulating CD206+ M2-like macrophage polarization
Source: Cell Death Dis. 2020 Feb 19;11(2):136. doi: 10.1038/s41419-020-2329-z (PMC7031231; doi:10.1038/s41419-020-2329-z)
Supplement: Supplementary file 1 — Supplementary figure legends [file 41419_2020_2329_MOESM1_ESM.docx]

**Supplementary figure legends**

**Supplementary Fig. S1 Microcystin-LR ameliorates FITC-induced mouse pulmonary fibrosis.** Mice were intratracheally instilled with a single dose of fluorescein isothiocyanate (FITC) (3.5 mg/kg) on day 0 and received microcystin-LR (20 μg/L) in drinking water starting on day 14. Mice were euthanized and samples were collected for analysis day 56 after receiving FITC treatment. **a** Schematic representation of experimental design. **b** Lung tissue sections were prepared and subjected to immunofluorescence scanning, H&E and Masson’s trichrome staining. Scale bar: 2000 μm and 100 μm (insets). **c** FITC-associated relative fluorescence intensity in mouse pulmonary tissues was assessed. The integrated optical density (IOD) was analyzed by Image-Pro Plus 6.0. n = 3 mice per group. **d, e** The development of lesions revealed by H&E (**d**) and Masson’s trichrome staining (**e**) was scored by pathologists blind to the study design. **P* < 0.05, ***P* < 0.01 determined by one-way ANOVA with Student-Newman-Keuls (S-N-K) post-hoc analysis (inflammation score: F_2,27_ = 6.582, *P* = 0.005; masson score: F_2,27_ = 6.618, *P* = 0.005). n = 10 mice per group. **f** Total protein was extracted from mouse pulmonary tissues and examined for the expression of collagen 1α1 by western blot.

**Supplementary Fig. S2 Microcystin-LR reduces the expression levels of TGF-β1 and αSMA** **in lung tissues of bleomycin-induced model rats.** Rats were treated as explained in Fig. 1. Pulmonary tissue sections were examined for the expression of TGF-β1 and αSMA using immunohistochemistry. Black arrows indicate TGF-β1**^+^** or αSMA**^+^** cells. Scale bars: 100 μm. **P* < 0.05 and ***P* < 0.01 determined by one-way ANOVA with S-N-K post-hoc analysis (TGF-β1: F_4,28_ = 32.736, *P* < 0.000; αSMA: F_4,28_ = 4.418, *P* = 0.007). n = 5 (bleomycin) or n = 7 (all the other treatments).

**Supplementray Fig. S3 The regimen of microcystin-LR for bleomycin-induced pulmonary fibrosis does not inhibit PP2A activity. a, b** Rats were treated as explained in Fig. 1. A protein phosphatase type 2A (PP2A) activity assay (means ± SEM) in rat pulmonary tissues was conducted. Saline group was used as control. Each dot represents measurement of one individual rat (**a**). The contents of free microcystin-LR in pulmonary and liver tissues following various treatments groups were measured using an ELISA kit. * *P* < 0.05, ** *P* < 0.01 was determined by paired Student’s *t* test (**b**). **c, d** Human normal liver cell L02, a cell line with susceptibility to microcystin-LR, was cultured for 48 hours with the indicated concentrations of microcystin-LR. Alterations in PP2A activity of L02 cells were measured. The activity is expressed as the percent of activity of PP2A in extracts from untreated cells. ** *P* < 0.01 were determined by one-way ANOVA with S-N-K post-hoc analysis, F_5,12_ = 66.729, *P* < 0.000 (**c**). L02 cell yield was monitored continuously up to 48 hours using the xCelligence real-time cell analyzer (RTCA). The cell indices were normalized to reflect the cell growth relative to the initial values (at the start of exposure) (**d**).

**Supplementary Fig. S4 Inhibitory effect of microcystin-LR on EMT and FMT is mediated by M2 macrophage modulation rather than M0 or M1.** **a** Mouse bone marrow derived macrophages were cultured for 48 hours with IL-4 (5 ng/ml) to induce the polarization of macrophages in 6-well Millicell hanging cell culture inserts with a membrane pore size of 1.0 μm. Some of the cells were also treated with 0.1 μM microcystin-LR as indicated. Next, cell culture inserts containing the pretreated macrophages were transferred to the plates that were seeded with A549, MRC5 or NIH3T3 cells 24 hours ago. After the coculture for 48 hours, the expression levels of various proteins as indicated in A549, MRC5 and NIH3T3 cells were examined by western blot. **b** RAW264.7 cells were left alone or cultured for 48 hours with LPS (10 ng/ml) to produce M0 or M1 macrophages. Some of the cells were also treated with 0.1 μM microcystin-LR as indicated. After the coculture with A549, MRC5 or NIH3T3 cells for 48 hours, the expression levels of EMT or FMT markers in A549, MRC5 and NIH3T3 cells were examined by western blot.

**Supplementary Fig. S5 Differential uptake of microcystin-LR by monocyte/macrophages, fibroblasts and epithelial cells *in vitro*.** RAW264.7, MRC5 and A549 were incubated with 0.1 μM microcystin-LR for 24 hours followed by immunofluorescent analysis detecting microcystin-LR (red) uptaken by the cells. Scale bar: 10 μm.

**Supplementary Fig. S6 Microcystin-LR has no significant effect on bleomycin-associated increase of CD68^+^ macrophages in the lung tissues.** Rats were treated as explained in Fig. 1. **a** Pulmonary tissue sections were examined for the expression of CD68, a macrophage marker, with immunohistochemistry. Scale bars: 100 μm. **b** Quantification of total CD68**^+^** cells. ***P* < 0.01 determined by one-way ANOVA with S-N-K post-hoc analysis (F_4,28_ = 13.719, *P* < 0.000). n = 5 (bleomycin) or n = 7 (all the other treatments).

**Supplementary Fig. S7 Microcystin-LR mainly suppresses anti-inflammatory factors rather than pro-inflammatory factors.** Rats were treated as explained in Fig. 1. mRNA was purified from pulmonary tissues and examined for the expression of anti-inflammatory molecules, including arginase-1 (Arg1; **a**), resistin-like α (Fizz1; **b**), chitinase-like 3 (Ym1; **c**) and IL-10 (**d**), and pro-inflammatory molecules including TNF-α (**e**), IL-6 (**f**), IL-1β (**g**) and NF-κB (**h**) by quantitative RT-PCR (qRT-PCR). The mRNA levels for the saline controls were normalized to 1. **P* < 0.05 and ***P* < 0.01 determined by one-way ANOVA with S-N-K post-hoc analysis (Arg1: F_4,28_ = 13.911, *P* < 0.000; Fizz1: F_4,28_ = 18.744, *P* < 0.000; Ym1: F_4,28_ = 2.732, *P* = 0.049). n = 5 (bleomycin) or n = 7 (all the other treatments).

**Supplementary Fig. S8 Microcystin-LR does not significantly affect the cell cycle and apoptosis of monocyte/macrophages *in vitro*.** RAW264.7 cells were left alone or treated with microcystin-LR (0.1 μM) for 48 hours. **a** Cell apoptosis was measured using Annexin V/propidium iodide (PI) double staining, followed by flow cytometry. The representative profiles on flow cytometry are shown (left panel). The mean percentage of Annexin V-positive cells are shown in the right panel. Data were analyzed using unpaired Student’s *t* test. n = 5. **b** Expression of caspase-3, cleaved caspase-3, poly(ADP-ribose) polymerase (PARP) and cleaved PARP was analyzed by western blot. **c** Representative flow cytometric analysis of the cell cycle distribution in RAW264.7 cells (left panel). Quantitative analysis of the cell cycle distribution results in right panel. Data were analyzed using unpaired Student’s *t* test. n = 3.

**Supplementary Fig. S9** **An overlapped localization of CD206 and GRP78 in the pulmonary resident cells of model rats.** Rats were treated as explained in Fig. 1. Rat pulmonary tissues were analyzed using immunofluorescence to reveal localization of GRP78 (green) with CD206 (red). Scale bar: 20 μm.

**Supplementary Fig. S10 Microcystin-LR does not significantly aggravate bleomycin-induced changes in peripheral blood counts.** Rats were treated as explained in Fig. 1. Whole blood was collected from abdominal aorta and examined for RBC (**a**), WBC (**b**), platelet (PLT; **c**), neutrophil (**d**), lymphocyte (**e**) and monocyte (**f**) counts by automatic blood analyzer. **P* < 0.05, ***P* < 0.01 determined by one-way ANOVA with S-N-K post-hoc analysis (WBC: F_4,20_ = 3.374, *P* = 0.029; neutrophil count: F_4,20_ = 3.195, *P* = 0.035; monocyte count: F_4,20_ = 4.649, *P* = 0.008). n = 7 (saline), n = 4 (bleomycin and LR7) or n = 5 (all the other treatments).

**Supplementary Fig. S11 Microcystin-LR does not significantly cause histological damage on liver and kidney tissues of the rats with bleomycin-induced pulmonary fibrosis.** Rats were treated as explained in Fig.1. Tissue sections of liver and kidneys were prepared and subjected to H&E staining. Scale bar: 200 μm.
