# Supplementary material for: Microcystin-LR ameliorates pulmonary fibrosis via modulating CD206+ M2-like macrophage polarization
Source: Cell Death Dis. 2020 Feb 19;11(2):136. doi: 10.1038/s41419-020-2329-z (PMC7031231; doi:10.1038/s41419-020-2329-z)
Supplement: Supplementary file 2 — Supplementary table 1 [file 41419_2020_2329_MOESM2_ESM.docx]

**Supplementary Table 1**. **Biochemical characteristics measurements of microcystin-LR treated rats.**

| **Treatment** | **Saline** | **Bleomycin** | **Bleomycin + LR7** | **Bleomycin + LR14** | **Bleomycin + LR28** |
| --- | --- | --- | --- | --- | --- |
| AST (UI/L)  Mean ± SD | 118.57 ± 41.15 | 113.75 ± 51.55 | 126.25 ± 25.95 | 125.00 ± 43.36 | 105.20 ± 14.58 |
| ALT (UI/L)  Mean ± SD | 39.14 ± 16.75 | 43.50 ± 26.34 | 40.00 ± 14.45 | 39.00 ± 9.38 | 37.00 ± 9.30 |
| CRE (mmol/L)  Mean ± SD | 82.86 ± 9.32 | 84.25 ± 11.59 | 97.00 ± 17.91 | 99.40 ± 24.95 | 96.80 ± 14.36 |
| BUN (mmol/L)  Mean ± SD | 5.70 ± 0.67 | 6.70 ± 1.28 | 6.38 ± 1.55 | 6.25 ± 1.59 | 5.96 ± 0.96 |
| TP (g/L)  Mean ± SD | 52.29 ± 2.98 | 52.25 ± 1.71 | 54.75 ± 3.69 | 54.00 ± 3.74 | 51.80 ± 1.92 |
| ALB (g/L)  Mean ± SD | 30.29 ± 0.76 | 30.75 ± 0.50 | 30.75 ± 1.50 | 30.40 ± 1.52 | 29.40 ± 1.14 |
| TG (mmol/L)  Mean ± SD | 1.34 ± 0.32 | 1.72 ± 0.60 | 2.20 ± 1.00 | 1.97 ± 1.11 | 1.51 ± 0.42 |
| TC (mmol/L)  Mean ± SD | 1.98 ± 0.34 | 1.83 ± 0.14 | 1.80 ± 0.33 | 1.96 ± 0.38 | 1.72 ± 0.23 |
| HDL (mmol/L)  Mean ± SD | 1.31 ± 0.17 | 1.23 ± 0.06 | 1.31 ± 0.26 | 1.10 ± 0.34 | 1.22 ± 0.11 |
| LDL (mmol/L)  Mean ± SD | 1.64 ± 0.31 | 1.52 ± 0.10 | 1.90 ± 0.37 | 2.25 ± 1.18 | 1.42 ± 0.22 |

Rats were treated as explained in Fig. 1. The serum was separated from whole blood collected from abdominal aorta and examined for the biochemical parameters as indicated. Data was analyzed by one-way ANOVA with S-N-K post-hoc analysis. LR, microcystin-LR; AST, aspartate aminotransferase; ALT, alanine aminotransferase; CRE, creatinine; BUN, blood urea nitrogen; TP, total protein; ALB, albumin; TG, triglycerides; TC, cholesterol; HDL, high density lipoprotein; LDL, low density lipoprotein. n = 4 (Bleomycin and Bleomycin + LR7) or n = 5 (all the other treatments).
