# Supplementary material for: Microcystin-LR ameliorates pulmonary fibrosis via modulating CD206+ M2-like macrophage polarization
Source: Cell Death Dis. 2020 Feb 19;11(2):136. doi: 10.1038/s41419-020-2329-z (PMC7031231; doi:10.1038/s41419-020-2329-z)
Supplement: Supplementary file 3 — Supplementary table 2 [file 41419_2020_2329_MOESM3_ESM.docx]

**Supplementary Table 2. Primers for qRT-PCR**

| **Genes** | **Sequences** |
| --- | --- |
| Rat IL-1β | F: GACTTCACCATGGAACCCGT |
|  | R: GGAGACTGCCCATTCTCGAC |
| Rat TGF-β1 | F: AGGGCTACCATGCCAACTTC |
|  | R: CCACGTAGTAGACGATGGGC |
| Rat Collagen 1α1 | F: GGAGAGAGCATGACCGATGG |
|  | R: GGGACTTCTTGAGGTTGCCA |
| Rat NF-κB | F: TTCAACATGGCAGACGACGA |
|  | R: CCATCTGTTGACAGTGGTATATCTG |
| Rat P4HA3 | F: GAGTACCGCATCAGCAAAAG |
|  | R: CCCTCCAATTCCATAGTTCAC |
| Rat GAPDH | F: AGTGCCAGCCTCGTCTCATA |
|  | R: GGTAACCAGGCGTCCGATAC |
| Rat αSMA | F: ACCATCGGGAATGAACGCTT |
|  | R: CTGTCAGCAATGCCTGGGTA |
| Rat Fibronectin | F: GGATCCCCTCCCAGAGAAGT |
|  | R: GGGTGTGGAAGGGTAACCAG |
| Rat TNF-α | F: CGGGCAGGTCTACTTTGGAG |
|  | R: TGGACCCAGAGCCACAATTC |
| Rat IL-6 | F: CTCTCCGCAAGAGACTTCCA |
|  | R: TCTCCTCTCCGGACTTGTGAA |
| Rat Smad3 | F: TGATTCTGCCACATCCCTGC |
|  | R: ACCCCTACCCTTTAGGGACC |
| Rat CD206 | F: TGATTCCGGTCGCTGTTCAA |
|  | R: GAACGGAGATGGCGCTTAGA |
| Rat CD163 | F: TCCGGTTGAAGTTTTGTGACC |
|  | R: GTGGTCCCGATGACCGTATT |
| Rat GRP78 | F: CTGTGAGACACCTGACCGAC |
|  | R: GACGCAGGAATAGGTGGTCC |
| Rat IL-10 | F: CAAAGAGAACGCGTGGAACG |
|  | R: CCAGGTTCGGTCGGAATAGG |
| Rat Arg1 | F: TTGGAACGAAACGGGAAGGT |
|  | R: TGTTCGGTTTGCTGTGATGC |
| Rat iNOS | F: AGAGACGCTTCTGAGGTTCC |
|  | R: CTGCACCAACTCTGCTGTTC |
| Rat Fizz1 | F: CGAGGGGACACTGACTTTCAA |
|  | R: CTGGGACCATCAGCTGGAGA |
| Rat Ym1 | F: ACCCCTGCCTGTGTACTCACCT |
|  | R: CACTGAACGGGGCAGGTCCAAA |
| Mu Ym1 | F: GCAAGACTTGCGTGACTATGAA |
|  | R: AACGGGGCAGGTCCAAA |
| Mu TGF-β | F: AGCTGCGCTTGCAGAGATTA |
|  | R: AGCCCTGTATTCCGTCTCCT |
| Mu Arg1 | F: CGGCAGTGGCTTTAACCTTG |
|  | R: TTGGGAGGAGAAGGCGTTTG |
| Mu CD206 | F: TAGCACTGGGTTGCATTGGT |
|  | R: TGCAGGGTTGACATGAGACC |
| Mu GAPDH | F: CCCTTAAGAGGGATGCTGCC |
|  | R: TACGGCCAAATCCGTTCACA |
