# Supplementary figures and images for: Microcystin-LR ameliorates pulmonary fibrosis via modulating CD206+ M2-like macrophage polarization
Source: Cell Death Dis. 2020 Feb 19;11(2):136. doi: 10.1038/s41419-020-2329-z (PMC7031231; doi:10.1038/s41419-020-2329-z)

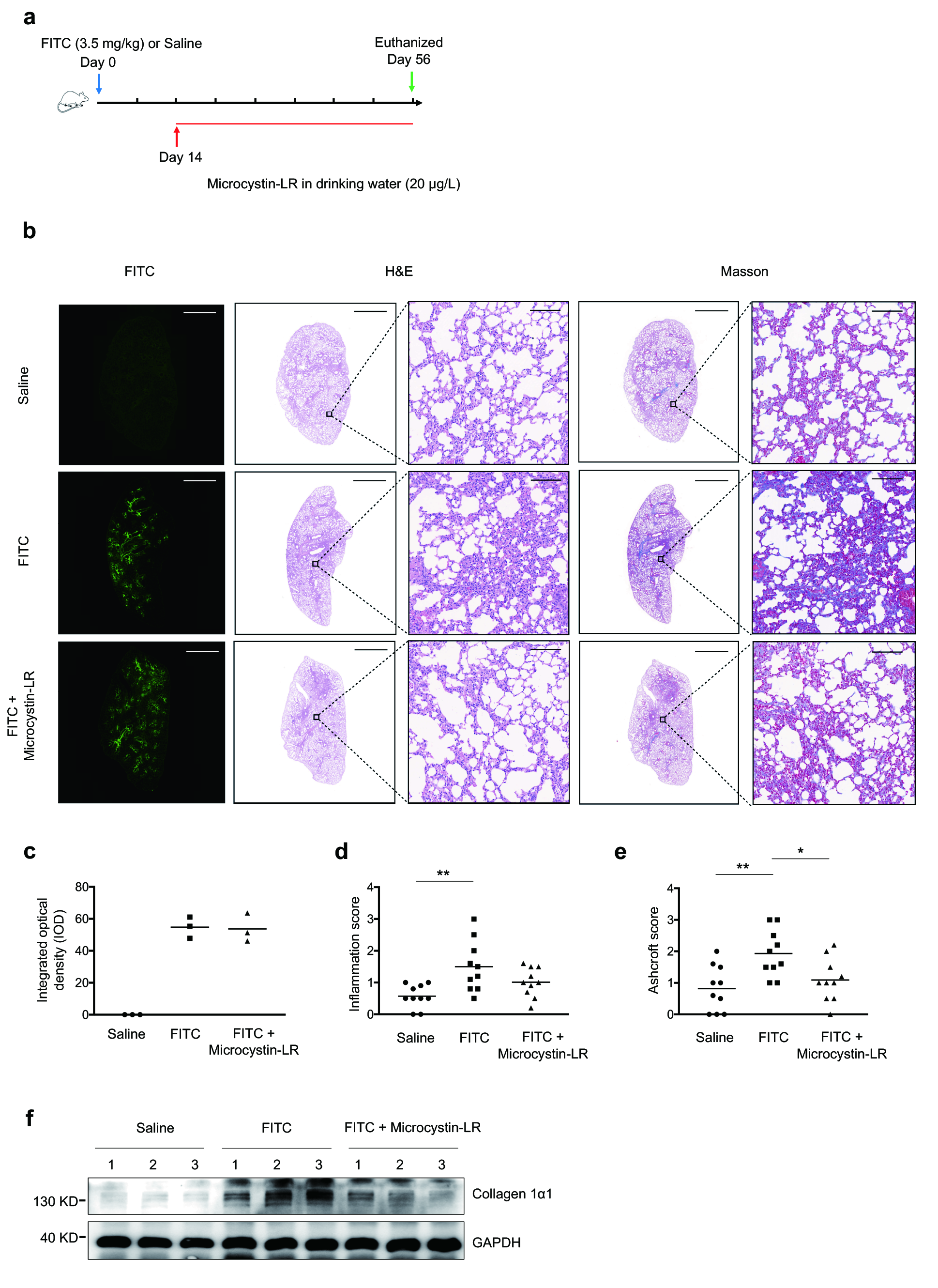

Supplement: Supplementary file 4 — Supplementary figure 1 [file 41419_2020_2329_MOESM4_ESM.tif]

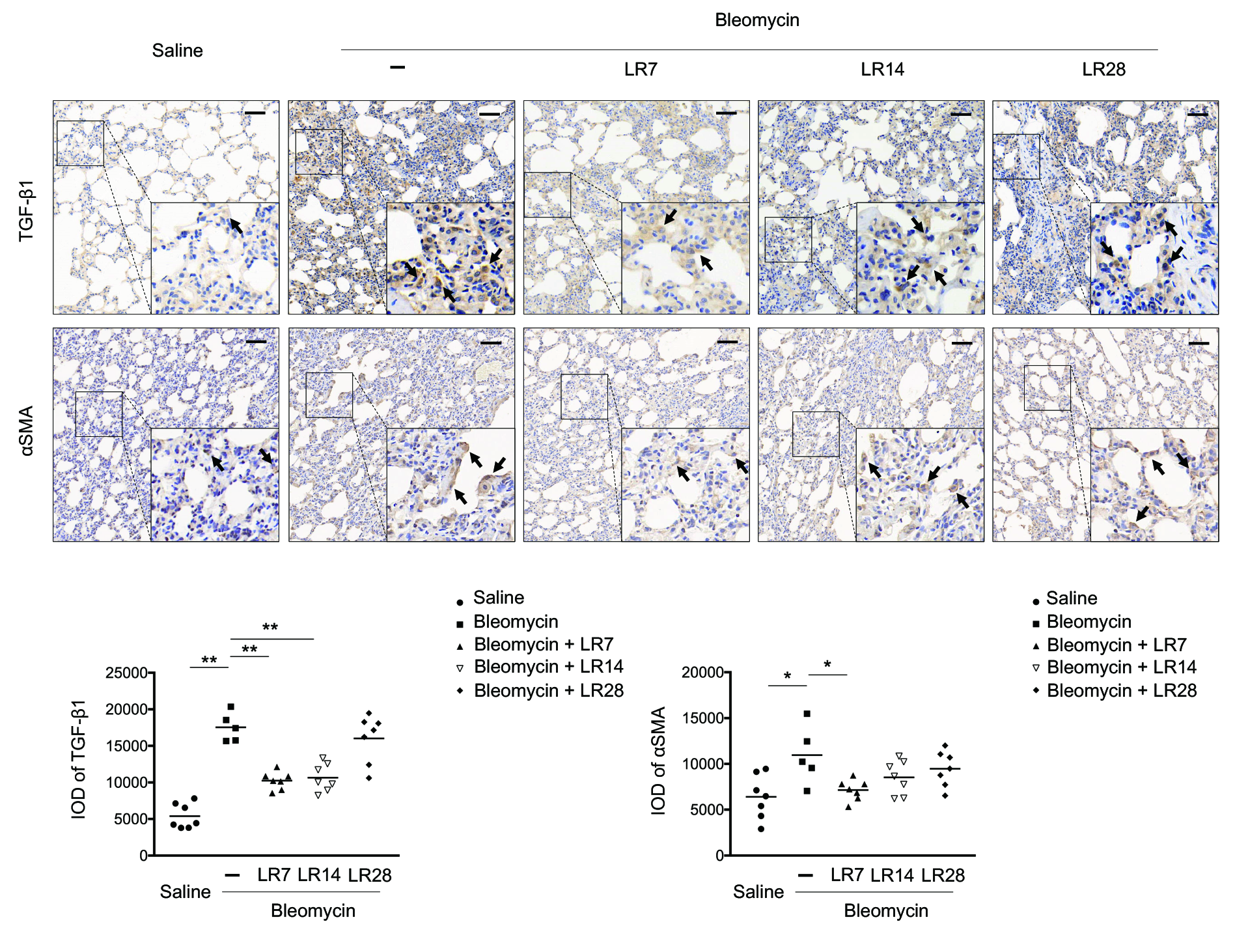

Supplement: Supplementary file 5 — Supplementary figure 2 [file 41419_2020_2329_MOESM5_ESM.tif]

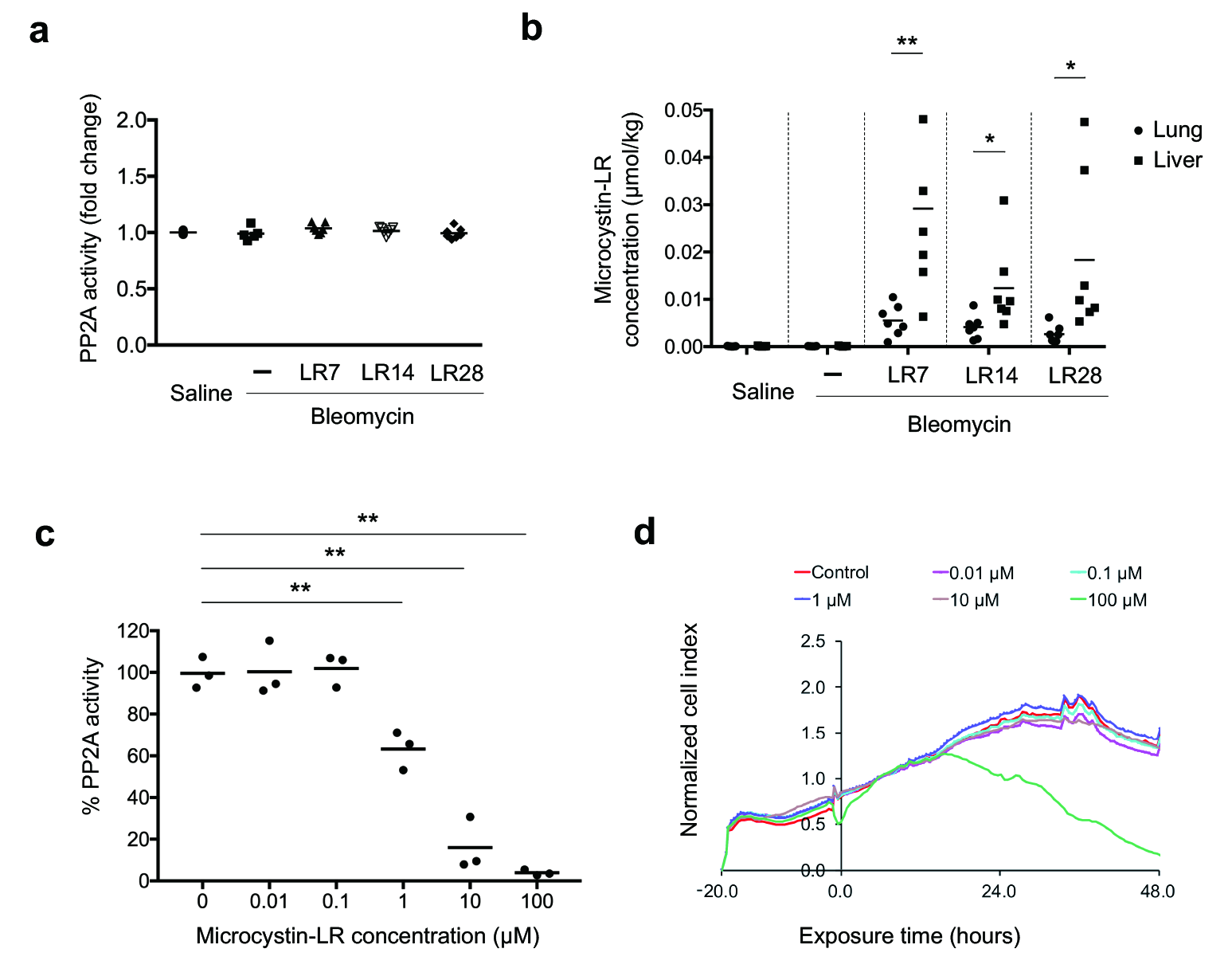

Supplement: Supplementary file 6 — Supplementary figure 3 [file 41419_2020_2329_MOESM6_ESM.tif]

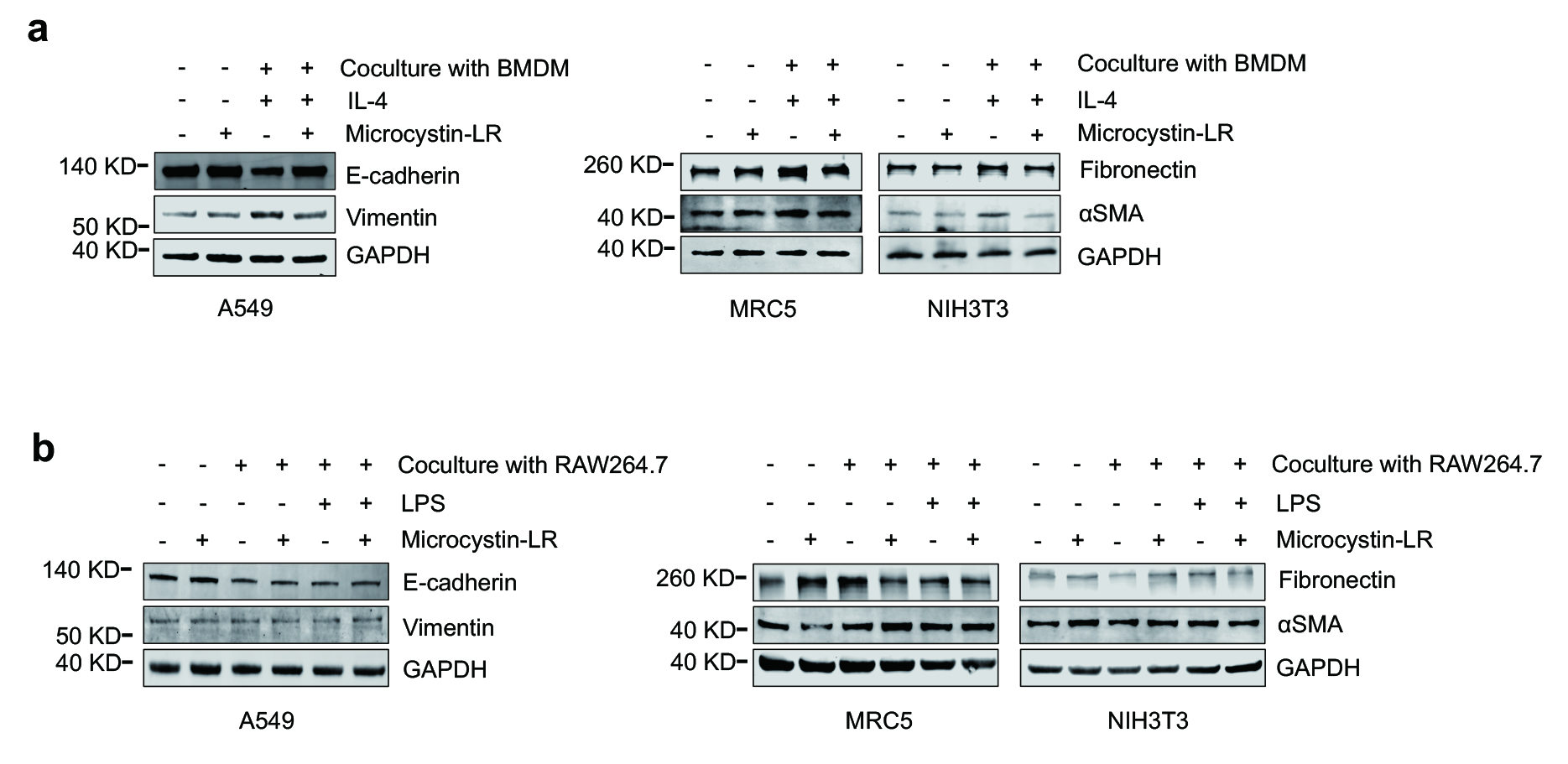

Supplement: Supplementary file 7 — Supplementary figure 4 [file 41419_2020_2329_MOESM7_ESM.tif]

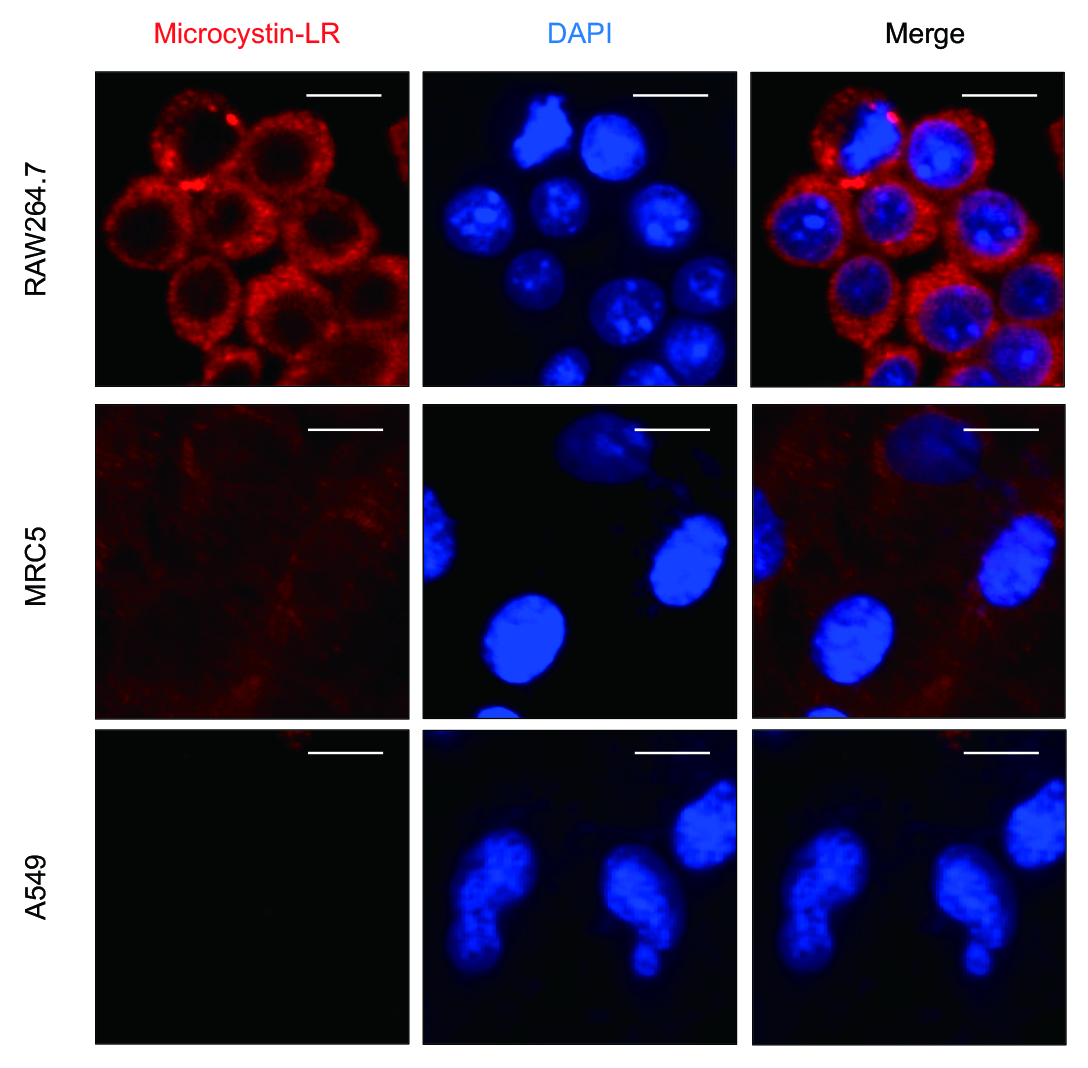

Supplement: Supplementary file 8 — Supplementary figure 5 [file 41419_2020_2329_MOESM8_ESM.tif]

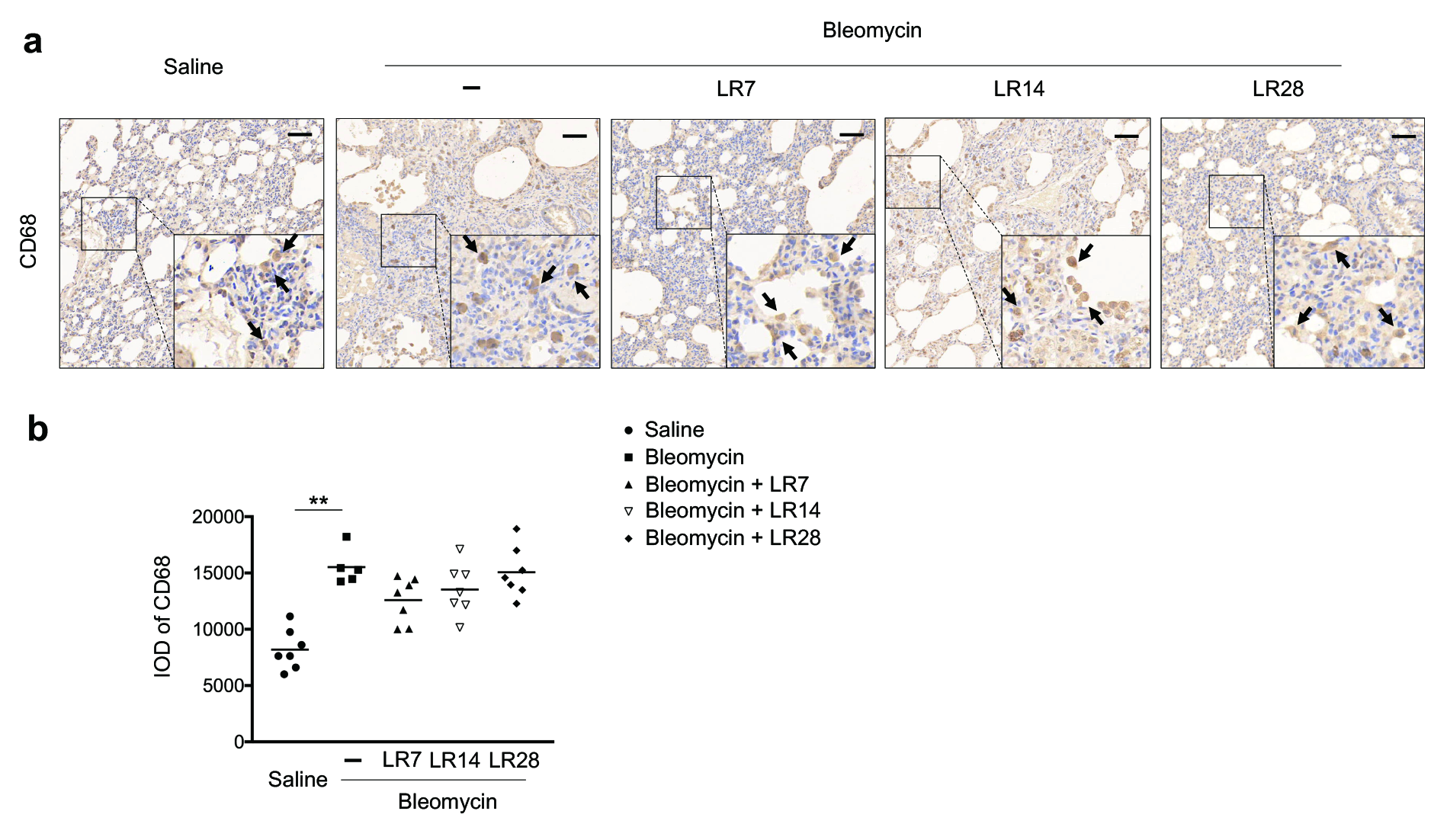

Supplement: Supplementary file 9 — Supplementary figure 6 [file 41419_2020_2329_MOESM9_ESM.tif]

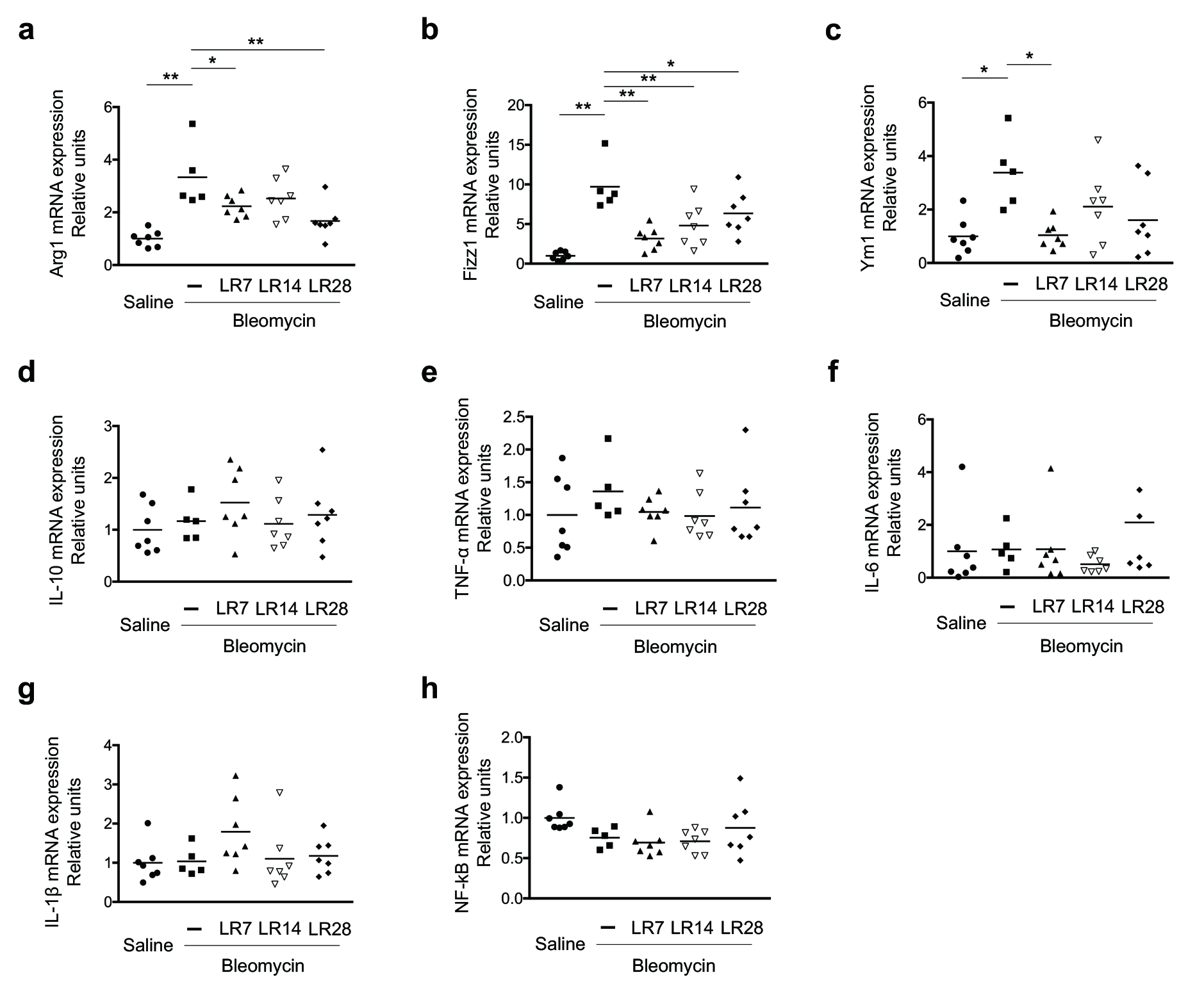

Supplement: Supplementary file 10 — Supplementary figure 7 [file 41419_2020_2329_MOESM10_ESM.tif]

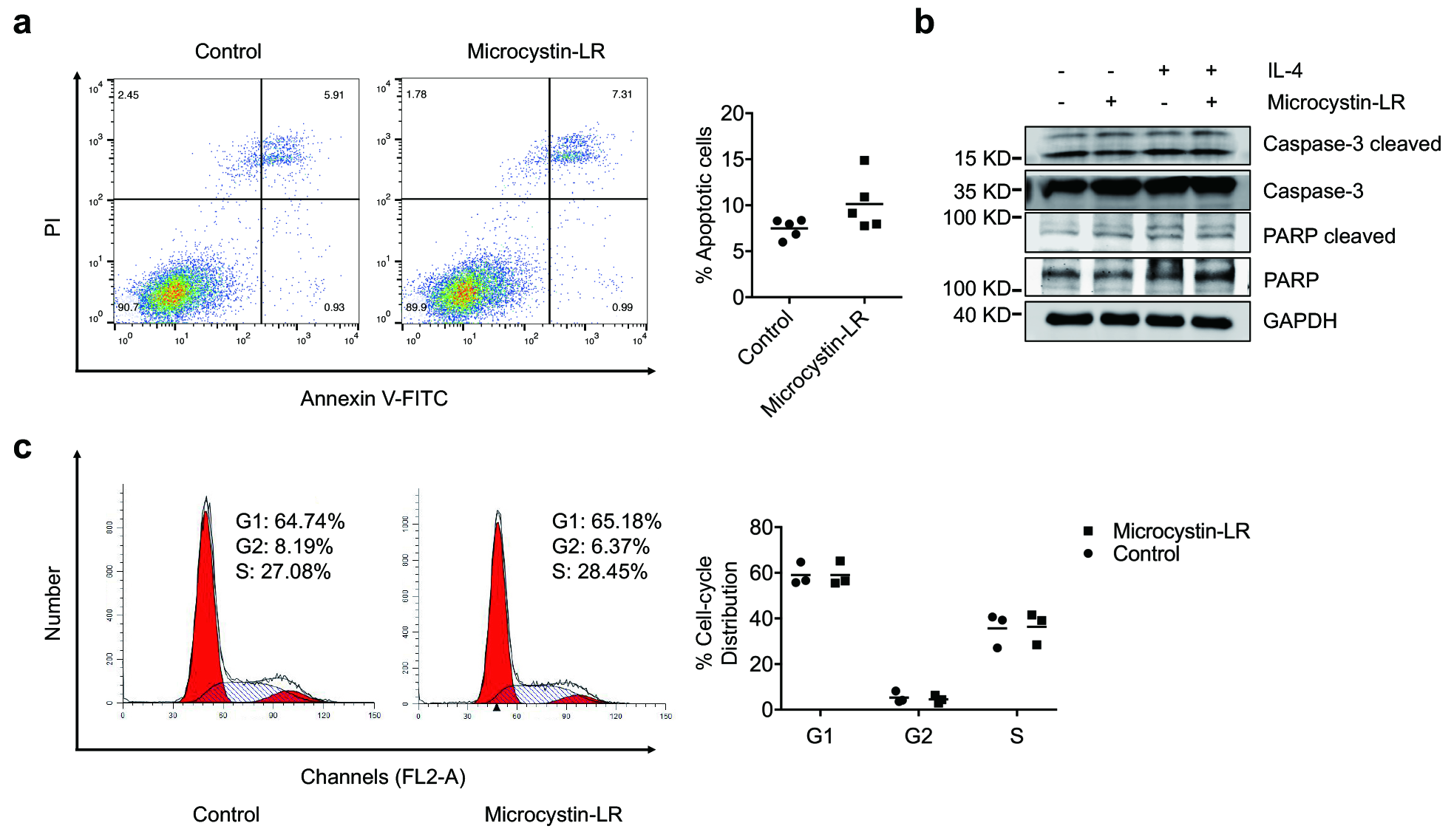

Supplement: Supplementary file 11 — Supplementary figure 8 [file 41419_2020_2329_MOESM11_ESM.tif]

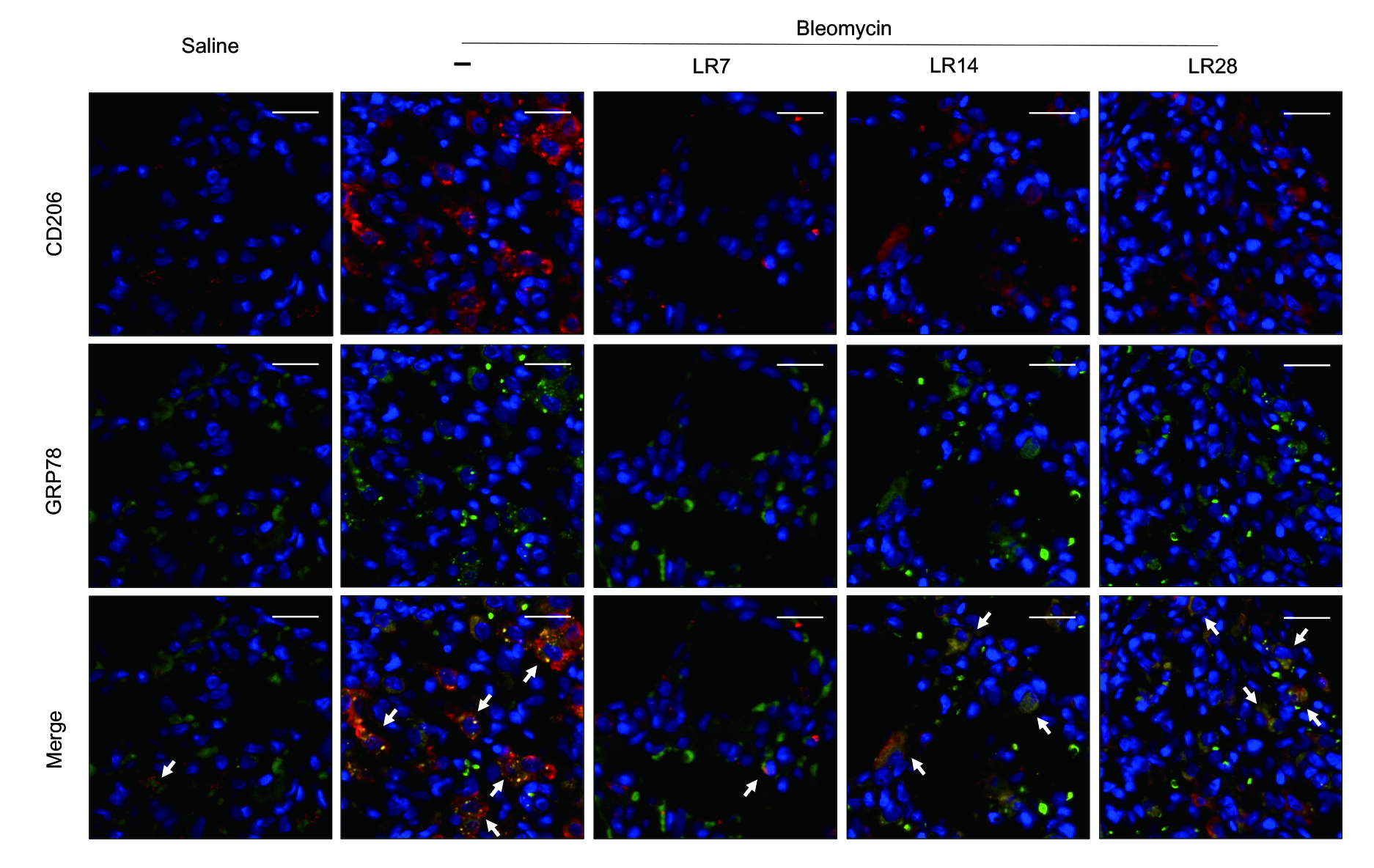

Supplement: Supplementary file 12 — Supplementary figure 9 [file 41419_2020_2329_MOESM12_ESM.tif]

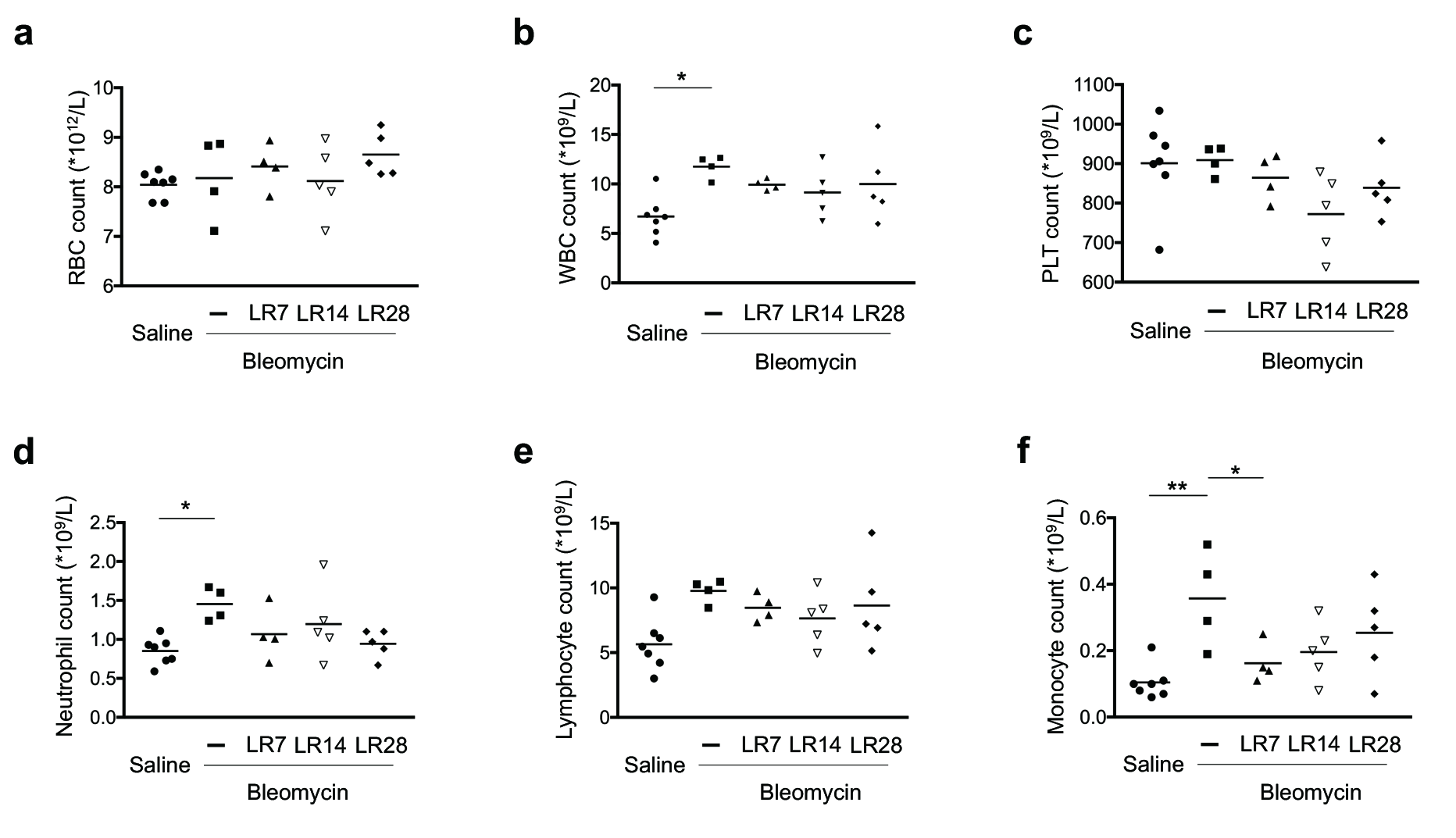

Supplement: Supplementary file 13 — Supplementary figure 10 [file 41419_2020_2329_MOESM13_ESM.tif]

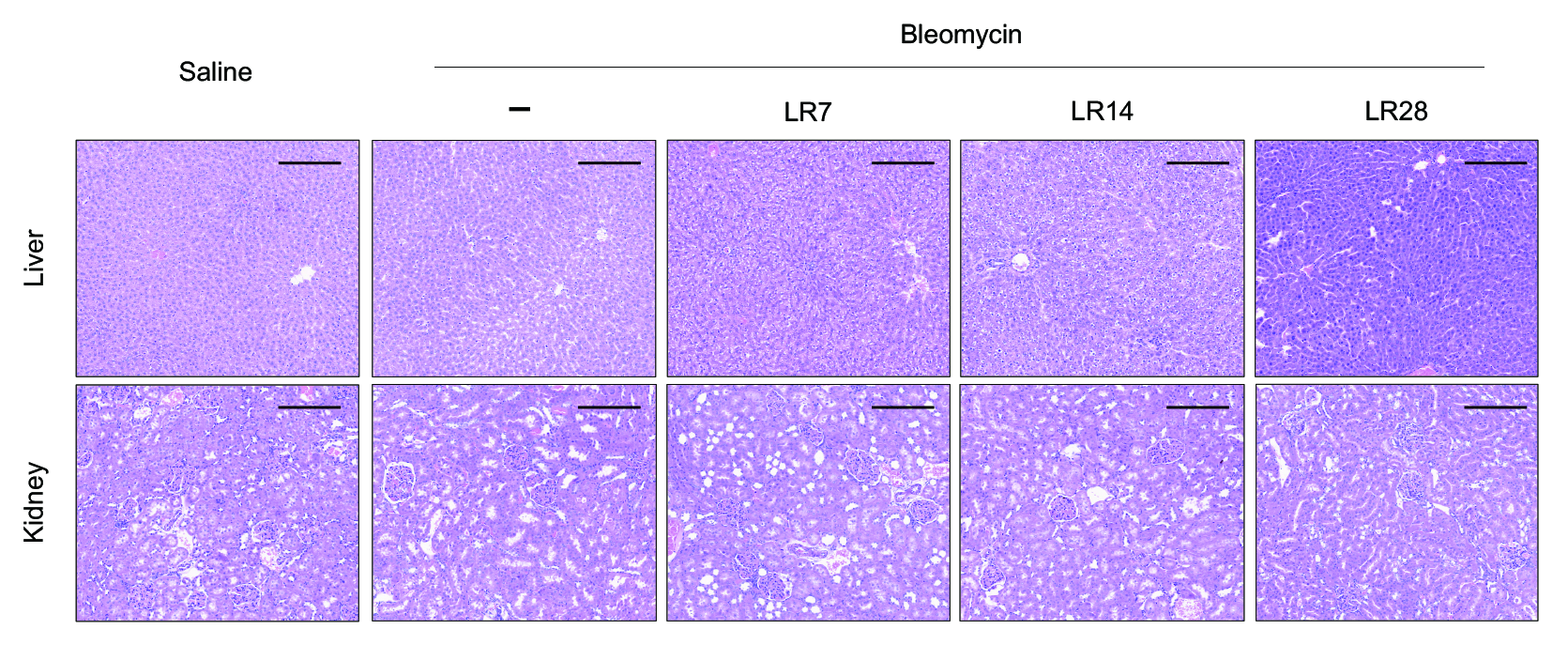

Supplement: Supplementary file 14 — Supplementary figure 11 [file 41419_2020_2329_MOESM14_ESM.tif]
